# Alternative splicing in ABA signaling during seed germination

**DOI:** 10.3389/fpls.2023.1144990

**Published:** 2023-03-16

**Authors:** Ewa Sybilska, Agata Daszkowska-Golec

**Affiliations:** Institute of Biology, Biotechnology and Environmental Protection, Faculty of Natural Sciences, University of Silesia in Katowice, Katowice, Poland

**Keywords:** alternative splicing, abscisic acid, seed germination, protein isoforms, splice variant, splicing factors, ABA signaling

## Abstract

Seed germination is an essential step in a plant’s life cycle. It is controlled by complex physiological, biochemical, and molecular mechanisms and external factors. Alternative splicing (AS) is a co-transcriptional mechanism that regulates gene expression and produces multiple mRNA variants from a single gene to modulate transcriptome diversity. However, little is known about the effect of AS on the function of generated protein isoforms. The latest reports indicate that alternative splicing (AS), the relevant mechanism controlling gene expression, plays a significant role in abscisic acid (ABA) signaling. In this review, we present the current state of the art about the identified AS regulators and the ABA-related changes in AS during seed germination. We show how they are connected with the ABA signaling and the seed germination process. We also discuss changes in the structure of the generated AS isoforms and their impact on the functionality of the generated proteins. Also, we point out that the advances in sequencing technology allow for a better explanation of the role of AS in gene regulation by more accurate detection of AS events and identification of full-length splicing isoforms.

## Role and molecular mechanism of alternative splicing

**Figure 1:**
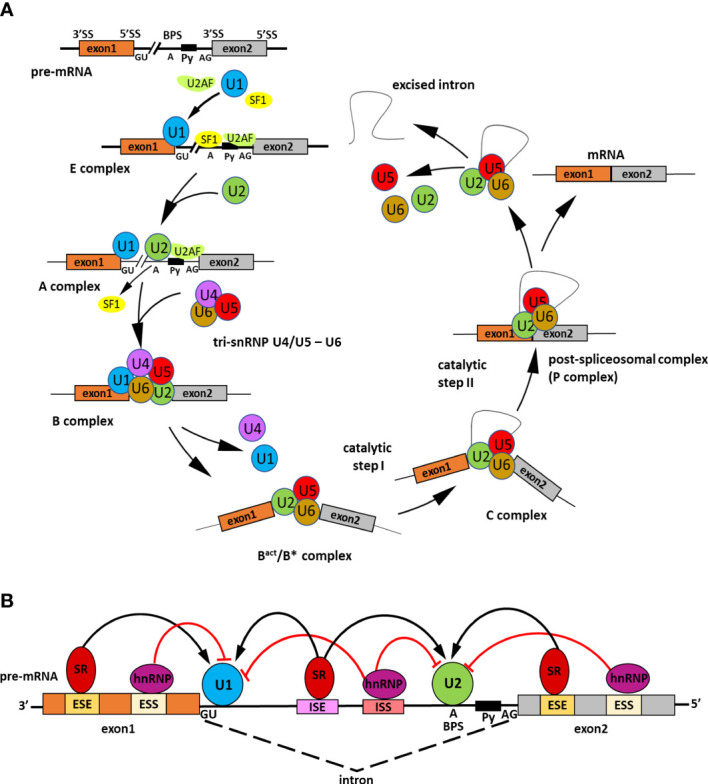
Scheme representing the splicing process and its regulation. **(A)** Steps of spliceosome assembly on precursor messenger RNA (pre-mRNA). Precise pre-mRNA splicing requires four constitutive motifs present in the intronic sequence: the 5′ splice site (GU), the 3′ splice site (AG), the branch point sequence (BPS), and the polypyrimidine tract (Py). The intron excision by the spliceosome is divided into several main stages. In the early step of splicing the E complex is generated. Firstly, splicing factor U1 snRNP U1 small nuclear ribonucleoprotein (U1 snRNP) attaches to the 5′ splice site. Next, SPLICING FACTOR 1 (SF1) recognizes the branch point sequence and U2 snRNP auxiliary factor (U2AF) in the polypyrimidine tract. The A complex is created when U2 snRNP displaces SF1. Then, tri-snRNP U4/U5-U6 combines with complex A to form an inactive B complex. The spliceosome is subjected to ATP-dependent structural changes and two U1 and U4 snRNPs detach leading to spliceosome catalytic activation and forming of an active B^act^/B^*^ complex. Next, intron excision is catalyzed in two subsequent transesterification reactions. The first reaction occurs in the C complex and the second in the C^*^complex. In the C^*^complex exons are joined together and as a result, is formed a post-spliceosomal complex with ligated exons (P complex). The splicing process is completed when the intron is twisted into a loop and excised; the exons are linked together to form mature mRNA, whereas snRNPs are recycled. **(B)** Role of *cis*-regulatory sequences and *trans*-acting splicing regulators in the spliceosome activity. The *cis*-regulatory sequences include intronic splice enhancers (ISEs) and silencers (ISSs), exonic splice enhancers (ESEs), and silencers (ESSs). To those sequences binds trans-acting RNA binding proteins (RBPs) which promote or inhibit splicing assembly. Serine/arginine (SR) proteins are activators of the splicing that bind ISEs/ESEs and promote exon joining through recruitment of U1 small nuclear ribonucleoprotein (snRNP) to the 5′ splice site and the U2 snRNP to the branch point. On the contrary, the heterogeneous nuclear ribonucleoproteins (hnRNPs) act as splicing inhibitors, attach to ISSs/ESSs, and repress splice site recognition (Gehring and Roignant, 2021).

**Figure 2:**
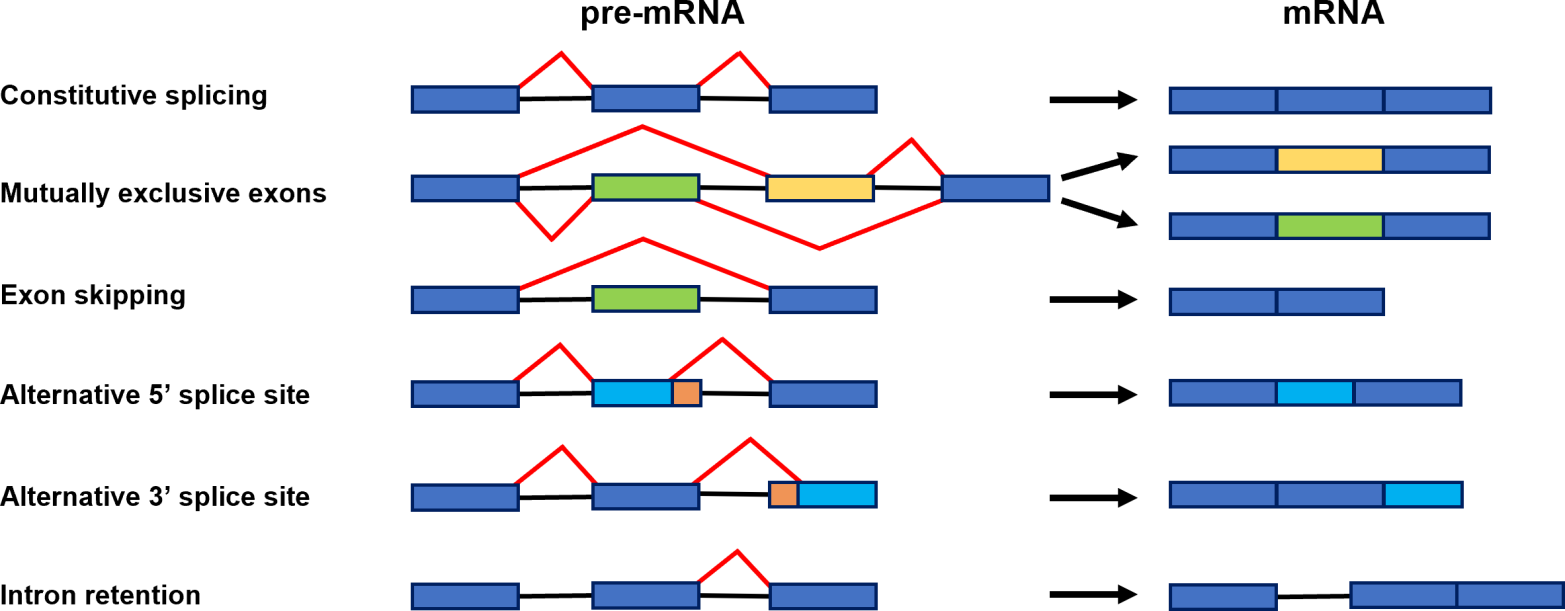
Scheme representing constitutive splicing and major alternative splicing events: constitutive splicing, mutually exclusive exons, exon skipping, alternative 5′ splice site, alternative 3′ splice site, and intron retention. Exons are shown as boxes and introns as black lines. Red lines illustrate distinct alternative splicing events (Gurnari et al., 2021).

Alternative splicing (AS) is a significant gene regulatory process in eukaryotic organisms. As a result of AS, many transcripts are produced from one gene, which can be translated into various proteins. The produced proteins can have similar or different functions in cells and vary in their properties (Verta and Jacobs, 2022). All of this makes AS contribute to increasing transcriptome and proteome diversity (Pan et al., 2008; Nilsen and Graveley, 2010). The splicing occurs in the nucleus during transcription to pre-mRNA and is catalyzed by the spliceosome. The spliceosome is a big complex composed of proteins and RNA. It builds five small nuclear ribonucleoproteins (snRNPs), namely U1, U2, U4, U5, and U6, and many other proteins required for the proper splicing process (Reddy, 2007; Gehring and Roignant, 2021). In two transesterification reactions catalyzed by splicosome, introns are removed, and exons are linked. In several steps, the spliceosome recognizes conserved sequences at exon-intron junctions and undergoes precise assembly and remodeling (Meyer et al., 2015) (**Figure 1A**). The spliceosome functions through numerous RNA and protein interactions and its activity is coordinated by multiple *cis*-acting elements and *trans*-acting factors (Punzo et al., 2020; Lin and Zhu, 2021) (**Figure 1B**). The constitutive and alternative splicing is distinguished. AS events can be grouped into five general splicing events such as intron retention (IR), exon skipping (ES), mutually exclusive exons, alternative 5′ donor sites (A5′SS) or alternative 3′ acceptor sites (A3′SS) (Kathare and Huq, 2021) (**Figure 2**). All introns are excised from pre-mRNA in constitutive splicing, and mature mRNA is assembled from exons. This produces one type of mRNA and generates only one type of protein. While in alternative splicing, introns and exons are removed or included from pre-mRNA in various ways, producing many mRNA isoforms. During exon skipping, the exon is removed from the pre-mRNA together with introns. The intron retention appears when both 5′ and 3′ ends of the specific intron are wrongly recognized, producing mRNA containing that additional intronic sequence. In mutually exclusive exons, only one exon from the cluster of exons remains in the mRNA, while the others are spliced out. When an alternative 5′ splicing site is generated, called a donor site as well, it causes cutting out of the fragment of exon from its 3′ end and nearby intron. The generation of an alternative splice site 3′, which is called the acceptor site as well, causes the cutting out of the intron with the 5′ fragment of the downstream exon (Syed et al., 2012; Chaudhary et al., 2019b). Estimating the number of AS events is demanding because AS is a dynamic process that changes quickly and depends on the type of the cell or tissue, the developmental stage, and environmental conditions (Chaudhary et al., 2019b). However, recent sequencing technologies allowed us much better to evaluate genes that are found to undergo AS. It is reported that around 95% of multiexon genes of humans and even up to 83% of plant intron-containing genes are subjected to AS (Pan et al., 2008). Plant genes are shorter than animals’ and contain fewer exons and shorter introns. These differences in the structure of genes may be the reason for the different number of AS events in plants and animals (Barbazuk et al., 2008; Chang et al., 2017). The most common event of AS in plants is intron retention. In the case of Arabidopsis, this mechanism accounts for approximately 45%–56% of all AS events. Surprisingly, in plants, only 8% of AS are exon skipping events, in contrast to animals, where this AS event is the most common and represents around 58% of all AS events (Marquez et al., 2012; Reddy et al., 2013; Chaudhary et al., 2019b). Proteins generated due to AS may have altered properties or acquire new biological functions, while losing functional domains results in non-functional proteins (Chaudhary et al., 2019a). The change in the proportion of the functional to non-functional isoforms fine-tunes the developmental processes and allows the plants quickly react to the changing environmental conditions (Kalyna et al., 2012; Ohtani and Wachter, 2019). Alternative splicing also regulates the cell’s transcripts level by associating nonsense-mediated mRNA decay (NMD). NMD is the degradation of transcripts containing a premature stop codon (PTC), which causes premature translation termination. The alternatively spliced mRNA isoforms may have a PTC in their nucleotide sequence and then truncated proteins are made from such transcripts. Usually, these proteins are non-functional. Therefore, those aberrant mRNAs are directed and destroyed in the NMD pathway (Drechsel et al., 2013; Nasif et al., 2017). AS has been shown to regulate gene expression through association with the microRNA (miRNA) biogenesis process (Li and Yu, 2021). miRNAs are small and non-coding RNA molecules whose length is around 21–25 nucleotides. They repress the expression of the genes by degrading or inhibiting mRNA translation (Bartel, 2004). AS may occur during primary miRNA transcript (pri-miRNA) processing to mature miRNA. This generates changes in the structure of the pri-miRNA. As a result, the number of functional miRNAs in the cell changes, affecting the expression level of their target genes (Bielewicz et al., 2013; Zhang et al., 2022a). In plants, AS controls the key developmental processes such as growth, circadian clock or flowering time, and adaptation to external conditions, including abiotic stress or pathogen attack (Muhammad et al., 2022). Increasing evidence indicates that AS also regulates seed germination by modulating abscisic acid (ABA) signal transduction (Zhang et al., 2016; Narsai et al., 2017). ABA is the main phytohormone, which through its antagonism with gibberellins (GA), inhibits the process of seed germination (Finch-Savage and Footitt, 2017; Sano and Marion-Poll, 2021). At the seed germination stage, the relevant genes from the ABA signaling pathway, transcription factors, and spliceosome components are alternatively spliced (**Table 1**).

**Table 1:** List of ABA-related alternatively spliced genes that regulate seed germination.

Gene name (ID gene)	AS isoforms	Description	Reference
*HAB1* (At1g72770)	*HAB1.2* *HAB1.1*	*HAB1.1* positively and *HAB1.2* negatively regulate seed germination. RBM25 is a potential key regulator of alternative splicing of *HAB1*. ACINUS directly interacts with *HAB1 in vivo* and is involved in its splicing.	Wang et al., 2015 Bi et al., 2021
*ABH1*(*CBP80*) (At2g13540)	*ABH1.1* *ABH1.2*	Alternative splicing of *ABH1* is directly mediated by ACINUS.	Bi et al., 2021
*ABI3* (At3g24650)	*ABI3-α* *ABI3-β*	*ABI3* splicing during seed germination is regulated by DRT111 and SUA that act in the same pathway. *ABI3* homologous in wheat, rice, pea, tomato, flax and alfalfa undergo alternative splicing resulting in isoforms with changed functions.	Sugliani et al., 2010; Punzo et al., 2020 McKibbin et al., 2002; Wilkinson et al., 2005; Fan et al., 2007; Gagete et al., 2009; Gao et al., 2013; Wang et al., 2018; Lalanne et al., 2021
ABI5 (At2g36270)	*AT2G36270.1 AT2G36270.2*	AS splicing of ABI5 is controlled by the splicing factor SKIP. *OsABI5* generates *OsABI5-1* and *OsABI5-2* isoforms with a different ability of binding with OsABI3.	Zhang et al., 2022b Zou et al., 2007
*PIF6* (At3g62090)	*PIF6-α PIF6-β*	*PIF6* is alternatively spliced in seeds and only *PIF6-β* controls germination potential.	Penfield et al., 2010
*PTB1* (At3g01150) *PTB2* (At5g53180)	*SPI* *SPII*	PTBs auto-regulate and cross-regulate their alternative splicing. PTB1 and PTB2 regulate alternative splicing of *PIF6* to control the germination of seeds.	Stauffer et al., 2010 Rühl et al., 2012
*DOG1* (At5g45830)	*DOG1-α* *DOG1-β* *DOG1-γ DOG1-δ* *DOG1-ϵ*	DOG1 enhances ABA signaling through its interaction and inhibition of AHG1 and AHG3.	Née et al., 2017
*SR45* (At1g16610)	*SR45.1 SR45.2*	SR45 regulates the signaling of glucose and ABA at early seedling growth. *SR45* displayed isoform diversity during seed germination. SR45 plays role in salt stress tolerance as a positive regulator. *SR45.1* isoform is essential for salt stress adaptation.	Carvalho et al., 2010 Narsai et al., 2017 Albaqami et al., 2019
*SR45a* (At1g07350)	*SR45a-1a* *SR45a-1b*	SR45a-1a and SR45a-1b negatively regulate response to salt stress at the germination stage.	Li et al., 2021

## The alternative splicing modulates ABA signaling components during seed germination

**Figure 3:**
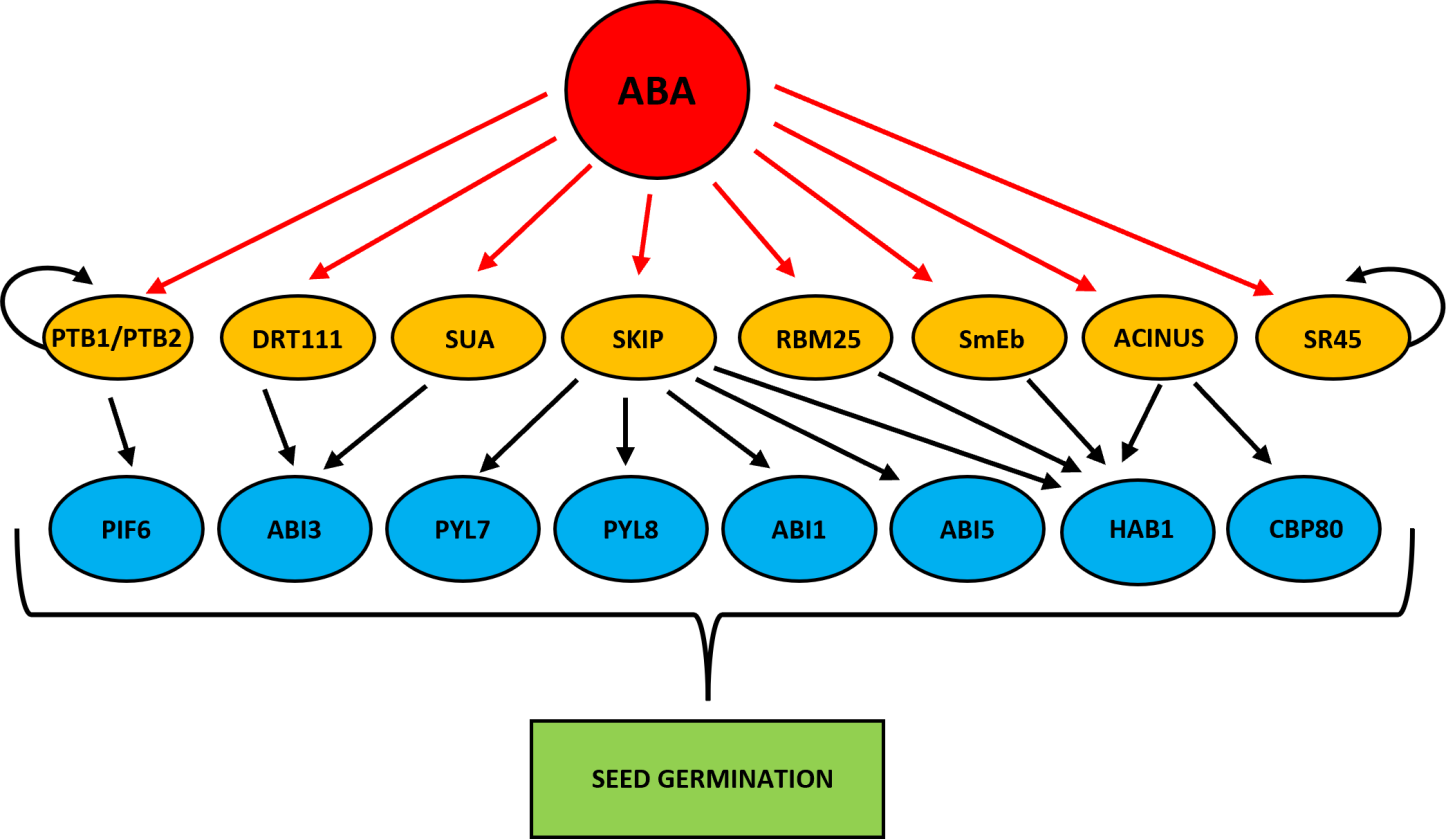
Scheme showing ABA-dependent splice factors that regulate alternative splicing of genes involved in the regulation of seed germination. Red arrows indicate ABA activation. Black arrows represent alternative splicing promotion. Curved arrows indicate autoregulation of alternative splicing.

HYPERSENSITIVE TO ABA1 (HAB1) phosphatase is one of the better described components of the ABA signaling pathway (Saez et al., 2004; Fujii et al., 2009). HAB1 binds and dephosphorylates SNF1-RELATED PROTEIN KINASE 2 (SnRK2) kinases, especially SnRK2.6 (OPEN STOMATA 1 (OST1), and inhibits the transduction of ABA signal (Umezawa et al., 2009; Vlad et al., 2009). The HAB1 belongs to the PP2C phosphatase family, but only in the case of *HAB1* the role of its AS isoforms at the germination stage is known (Wang et al., 2015). As a result of AS of *HAB1*, two protein isoforms are formed that differ in length. The *HAB1.1* is four-exonic, and its translation produces a full-length protein. The second isoform, *HAB1.2* is created due to intron retention between the third and fourth exon, and this intron sequence contains PTC. This variant produces a truncated protein with a partial catalytic domain because of lacking 105 amino acids at the C-terminal end. This defection changes the phosphatase activity of these two AS variants of *HAB1*. The *HAB1.1* and *HAB1.2* can bind to SnRK2, but only the long isoform *HAB1.1* can inactivate it by dephosphorylation, which prevents further ABA signal transduction. Notably, the expression level of both *HAB1* variants is high in seeds, but what is important is that the regulation of seed germination in the presence of ABA depends on the *HAB1.2*/*HAB1.1* isoform ratio. A decrease in the level of *HAB1.1* transcripts and an increase in *HAB1.2* turn on the ABA signal transduction. On the contrary, an increase in *HAB1.1* and a reduction in *HAB1.2* turn off the ABA pathway. Studies during seed germination in the presence of ABA using Arabidopsis mutants revealed the role played by *HAB1* isoforms in this process. The *hab1-1* insertional mutant is hypersensitive to ABA. However, the overexpression of *HAB1.1* in the *hab1-1* background leads to an ABA-insensitive phenotype, and transgenic seeds can germinate, in contrast to *hab1-1*. On the other hand, overexpression of *HAB1.2* in the *hab1-1* background does not complement the germination phenotype to the level of WT. All this indicates that those two *HAB1* variants generated in the AS process act antagonistically during seed germination. The role of *HAB1.1* is to promote germination, whereas the role of *HAB1.2* is to inhibit it (Wang et al., 2015). Studies with Arabidopsis mutants in the genes that encode the splicing factors RNA binding motif protein (RMB25), small nuclear ribonucleoprotein E (SmEb), SNW/Ski-interacting protein (SKIP), and ACINUS revealed their involvement in the regulation of AS *HAB1* (Cheng et al., 2017; Bi et al., 2021; Hong et al., 2021; Zhang et al., 2022b) (**Figure 3**). Mutants in these genes are hypersensitive to ABA during seed germination and show an altered expression pattern of *HAB1.1* and *HAB1.2* isoforms. The RNA binding motif protein (RMB25) contains PWI and RRM motifs important in RNA metabolism and splicing (Zhan et al., 2015). RMB25 interacts with other components of the splicing machinery and controls proper exon junctions (Carlson et al., 2017). ABA activates *RBM25* gene expression, and its translation product, the RBM25 protein, attaches to the last intron in the pre-mRNA of *HAB1* and generates two *HAB1* isoforms, *HAB1.1* and *HAB1.2*. The loss of function of RBM25 increases the *HAB1.2*/*HAB1.1* isoform ratio in the *rbm25* mutant compared to WT (Wang et al., 2015; Cheng et al., 2017). The SmEb protein is one of the seven Sm core proteins (SmB, SmD3, SmE, SmF, SmG, and SmD1) that are part of the pre-mRNA assembly machinery (Reddy, 2007; Schwer et al., 2016). SmEb contributes to regulating ABA signaling during seed germination through alternative splicing of HAB1 and activating ABA-positive regulators such as the transcription factors ABI3, ABI4, and ABI5 (Hong et al., 2021). Studies using *smeb-1* mutants have identified five *HAB1* gene transcripts (*HAB1.1*, *HAB1.2*, *HAB1.3*, *HAB1.4*, *HAB1.5*) from which only two versions of this protein are produced (Hong et al., 2021). Full-length HAB1.1 proteins are produced from *HAB1.1*, *HAB1.3*, and *HAB1.4* transcripts. In contrast, the truncated HAB1.2 protein is formed from *HAB1.2* and *HAB1.5*. After ABA treatment, the transcript levels of *HAB1.1*, *HAB1.3*, *HAB1.4*, and *HAB1.5* decreased in *smeb-1* mutants, but *HAB1.2* increased, resulting in decreased accumulation of the HAB1.1 protein isoform and increased accumulation of HAB1.2 protein. Additionally, only the overexpression of *HAB1.1* in the *smeb-1* background reversed the hypersensitive phenotype of the *smeb-1* mutant to ABA, while overexpression of *HAB1.2* did not restore the WT phenotype. This confirms that the AS *HAB1.1* variant is the functional HAB1 isoform regulating seed germination. Other studies using a mutant in the *SKIP* gene showed that apart from regulating *HAB1* splicing, it also directly regulates AS of other genes of the core ABA signaling pathway, such as PYRABACTIN RESISTANCE-LIKE 7 (PYL7), PYRABACTIN RESISTANCE-LIKE 8 (PYL8), ABA INSENSITIVE 1 (ABI1), and ABI5 (Zhang et al., 2022b). In response to ABA in the *skip-1* mutant, the number of transcripts of functional isoforms of positive ABA regulators PYL7, PYL8, PYL10, ABSCISIC ACID RESPONSIVE ELEMENT-BINDING FACTOR 1 (ABF1), ABF2, and ABI5 are reduced. On the other hand, the level of incorrect intron retention splicing form in negative regulators such as ABI1 and HAB1 is increased. Significantly, SKIP has previously been shown to interact with the SERINE/ARGININE-rich 45 (SR45) spliceosomal protein and control the circadian cycle through the AS of circadian clock genes (Wang et al., 2012). SKIP also controls the flowering time of plants through the AS and is responsible for plant tolerance to salt stress (Feng et al., 2015; Cui et al., 2017). Recently, the homolog of the mammalian apoptotic inducer of nuclear chromatin condensation (ACINUS) was identified in Arabidopsis (Bi et al., 2021). ACINUS, together with the serine-rich domain RNA-binding protein 1 (RNPS1) and the Sin3A-related protein 18 (SAP18), forms a complex of proteins associated with apoptosis and splicing (ASAP) (Schwerk et al., 2003). ACINUS contains an RNA recognition motif (RRM) required for RNA binding. A similar motif present in ACINUS in Arabidopsis has been identified in its distant paralog, another splicing factor called PININ (PNN). Through this motif, PININ also binds to RNPS1 and SAP18 and forms an alternative PSAP complex (Murachelli et al., 2012). Moreover, these two splice factors are also components of the exon junction complex (EJC), which is deposited onto pre-mRNAs to regulate the export of mRNA, translation, and NMD process (Tange et al., 2005; Schlautmann and Gehring, 2020). Research in Arabidopsis showed that ACINUS and PININ are redundant negative regulators of ABA responses. Knock-out of both *ACINUS* and *PININ* results in an abnormal plant phenotype. Double mutant *acinus-2 pinin-1* is dwarf and exhibits higher ABA sensitivity and delayed germination and flowering time dynamics (Bi et al., 2021). Transcriptomic analysis of the *acinus-2 pinin-1* double mutant exhibited ACINUS and PININ function in the AS mainly by promoting IR events. This type of AS was identified in *HAB1* pre-mRNA. In the mutant seedlings, non-functional *HAB1.2* was over-accumulated, while the short *HAB1.1* isoform was downregulated (Bi et al., 2021). Moreover, not spliced out intron has also been identified in another ABA negative regulator, the *ABA HYPERSENSITIVE 1/CAP BINDING PROTEIN 80 (ABH1/CBP80)* gene. ABH1/CBP80 and CBP20 are creating the CBC complex (Hugouvieux et al., 2001). Mutations in these genes confer ABA hypersensitive phenotype during seed germination (Hugouvieux et al., 2001; Hugouvieux et al., 2002; Papp et al., 2004; Jäger et al., 2011; Daszkowska-Golec et al., 2013; Daszkowska-Golec et al., 2017). The CBC complex co-transcriptionally interacts with the 5′-end cap structure of pre-mRNA and plays the role of an important splicing regulator (Laubinger et al., 2008; Raczynska et al., 2010). In plants, it has been shown that CBC together with the SERRATE (SE) protein promotes the interaction between the U1 small nuclear ribonucleoprotein (U1 snRNA) factor with the donor splice site of the first intron in the pre-mRNA (Laubinger et al., 2008). Studies with the use of single *cbp20* and *cbp80(abh1)* mutants as well as *cbp20/80* double mutant in Arabidopsis showed that a mutation in the *CBP80* gene generates more AS events than mutation in *CBP20*, which suggests that larger subunit CBP80 may play a decisive role in AS (Raczynska et al., 2010). In the *acinus-2 pinin-1* double mutant, the intron-containing isoform of *ABH1.2*, which produces a non-functional protein, was significantly accumulated, while the splicing form of *ABH1.1* was decreased (Bi et al., 2021). Together, all these studies indicate that the observed ABA-hypersensitive phenotype of the mutants in the *RMB25*, *SmEb*, *SKIP*, and *ACINUS* may relate to the accumulation of defective isoforms of ABA-negative regulators such as HAB1. Thus, these splice factors turn on or off the ABA signal transduction to fine-tune the seed germination by regulating the functional AS isoforms level in the cell.

## Transcription factors that regulate seed germination undergo ABA-related alternative splicing

AS regulates gene expression by producing various TF isoforms with altered or switched-off DNA binding abilities. This can influence the transcription activation of specific genes and change the transcription rate. One of the better known TFs that undergo AS in plants is ABSCISIC ACID INSENSITIVE 3 (ABI3)/VIVIPAROUS 1 (VP1). ABI3 plays a significant role in the maturation and germination of the seeds and positively modulates ABA signaling by activating ABA-responsive genes (Lopez-Molina et al., 2002; Finkelstein et al., 2008). Expression of *ABI3* is seed-specific and induced by ABA (Parcy et al., 1994; Liu et al., 2013). In Arabidopsis, seeds of *abi3* mutant are insensitive to ABA during germination and highly germinate, indicating that ABI3 negatively regulates seed germination in the presence of ABA (Finkelstein et al., 2008). ABI3 induces the expression of *MIR159*, which encodes the small non-coding RNA miR159. miR159 can regulate ABA signal transduction by targeting two transcription factors MYB DOMAIN PROTEIN 33 (MYB33) and MYB DOMAIN PROTEIN 101 (MYB101), which act as positive regulators of ABA signaling (Reyes and Chua, 2007). Moreover, ABI3 regulates the expression of *ABI4* and *ABI5*, which results in transcription activation of *LATE EMBRYOGENESIS ABUNDANT 1* (*EM1*) and *LATE EMBRYOGENESIS ABUNDANT 6* (*EM6*) genes and leads the inhibition of seed germination (Söderman et al., 2000; Lopez-Molina et al., 2002). Recently, it has been shown that ABI3 can also promote seed dormancy by inducing ABA biosynthesis (Liu et al., 2020). ABI3 binds to the promoter of the *REVERSAL of RDO5 1* (*ODR1*), which is a regulator of ABA biosynthesis, and inhibits its expression. This disrupts the interaction of the bHLH57 transcription factor with ODR1 and as a result, allows bHLH57 to activate the transcription of genes from the ABA biosynthetic pathway, such as *9-CIS-EPOXYCAROTENOID DIOXYGENASE 6* (*NCED6*) and *9-CIS-EPOXYCAROTENOID DIOXYGENASE 9* (*NCED9)* (Liu et al., 2020). However, as well alternative splicing of *ABI3* may affect the seed germination process by altering the function of this protein. AS of *ABI3* was detected in monocots and dicot plants (McKibbin et al., 2002; Fan et al., 2007; Gagete et al., 2009). ABI3 has four conserved and functional domains in its structure (A1 and B1, B2, B3) (Bies-Etheve et al., 1999; Kurup et al., 2000). The acidic A1 domain is located at the N-terminus and activates gene transcription (McCarty et al., 1991). The B1 domain is necessary for ABI3 interaction with other basic leucine zipper (bZIP) family transcription factors, like ABI5, bZIP10, bZIP25, and TRAB1 (Hobo et al., 1999; Ezcurra et al., 2000; Nakamura et al., 2001; Lara et al., 2003 Llorca et al., 2014). In the B2 domain, there is a nuclear localization signal. This domain is also required to transactivate genes containing ABA-responsive elements (ABRE) or G-box motifs (Ezcurra et al., 2000). The B3 domain has DNA binding activity and binds an RY motif (CATGCA), specific for the promoters of genes expressed in the seeds (Suzuki et al., 1997). For the first time, AS of *ABI3* was examined in pea (*Pisum sativum*) (Gagete et al., 2009). In this research were found seven transcripts of *PsABI3* the expression of which was detected during seed development, with the highest level mainly in the middle part of the embryogenesis process. The full-length PsABI3 protein containing all functional domains is generated from the *PsABI3-1* transcript. Functional PsABI3 protein is also produced from the *PsABI3-3* variant, which lacks a fragment located between the A1 and A2 acidic domains. This region contains many amino acids, i.e., proline, serine, and threonine, but its loss does not affect the activity of PsABI3. The PsABI3-1 and PsABI3-3 isoforms can interact with ABI5 due to the presence of the B1 domain. In the case of the *PsABI3-2* transcript, the A2 domain, the region between the two acidic domains, and a fragment of the B1 domain are missing, which generates an inactive protein incapable of interacting with ABI5. However, the presence of the B3 domain in PsABI3-2 may cause the ability of this protein to compete with active PsABI3-1 and PsABI3-3 in binding to promoters of target genes and thus prevent transcription of these genes. The PsABI3-5 protein is active despite having only the A1 domain but with an added short amino acid sequence. Notably, the presence of the A1 domain is insufficient for the biological activity of PsABI3. For this reason, the additional amino acid sequence is likely to cause the activity of the PsABI3-5 protein. The remaining three *PsABI3-4*, *PsABI3-6*, and *PsABI3-7* transcripts produce truncated proteins because of containing a premature termination codon (PTC). Those proteins cannot bind to the promoter sequence of target genes due to the loss of the B3 domain (Gagete et al., 2009). In the model plant Arabidopsis, two *ABI3* transcripts have been identified: *ABI3-α* and *ABI3-β*. The pre-mRNA of *ABI3-α* contains a cryptic intron which in translation produces a full-length protein with four functional domains, A1, B1, B2, and B3, which are important for DNA binding. The lack of a cryptic intron in the pre-mRNA of *ABI3-β* causes the translated protein not to have the B2 and B3 domains and therefore is defective. Analysis of the expression pattern of these AS isoforms revealed that both are expressed in seeds during their development. An increased level of *ABI3-α* transcript is observed during the entire seed development process. In contrast, an increase in the expression of the *ABI3-β* isoform is visible in the final stage of seed maturation (Sugliani et al., 2010). In Arabidopsis, it is known that alternative splicing of *ABI3* is regulated by the conserved splicing factor SUPPRESSOR OF ABI3-5 (SUA) (Sugliani et al., 2010). SUA is a homolog of the human RNA BINDING MOTIF PROTEIN 5 (RBM5), which binds to the splicing factor U2AF 65 and is responsible for 3′ splice site recognition (Bonnal et al., 2008). The same in Arabidopsis, SUA also interacts with the U2AF 65 subunit and, inhibiting excising of the cryptic intron, promotes the accumulation of the functional ABI3-α isoform. However, the *sua-1* mutant has a relatively high level of *ABI3-α* transcripts indicating that other splicing factors can replace the function of the SUA in AS *ABI3* during seed maturation (Sugliani et al., 2010). In the case of tomato (*Solanum lycopersicum*), the functional ABI3 protein is formed from the *S1ABI3-F* pre-mRNA (Gao et al., 2013). However, the absence of 90 bp in the pre-mRNA near the region encoding the functional B1 domain results in the formation of a truncated SlABI3-T protein. Interestingly, although *S1ABI3-T* has all four conserved ABI3 domains, its functioning is disturbed, suggesting the region’s important role near the B1 domain for the interaction with ABI5. Only the functional SlABI3-F isoform overexpression in tomato seeds causes hypersensitivity to ABA. This may be caused by the shortening of the *SlABI3-T* isoform close to the functional B1 domain, which results in a lower binding ability with ABI5 and consequently affects the efficiency of transcription of the ABI5 target genes and disrupts the ABA signaling. Both *S1ABI3* transcripts are upregulated mainly during seed imbibition, with the expression of the *S1ABI3-F* isoform significantly predominant at the germination stage. However, the use of various phytohormones, as well as abiotic stresses, changed the accumulation ratio of the shortened and full-length isoforms. Also, the expression of seed-specific ABA-dependent genes *SOMNUS* (*SlSOM*), *LATE EMBRYOGENESIS ABUNDANT 1* (*SlEM1*), and *LATE EMBRYOGENESIS ABUNDANT 6* (*SlEM6*) is regulated differently by both isoforms. The S1ABI3-F increases and S1ABI3-T reduces their expression. Interestingly, studies using transgenic plants with ectopic overexpression of *S1ABI3* isoforms in leaves have shown that, in this case, both isoforms function similarly and induce the expression of these ABA-dependent seed specific genes in the leaves. It indicates that the function of S1ABI3-F and S1ABI3-T depends on the tissue in which they are expressed (Gao et al., 2013). The AS of *ABI3* has also been shown in flax (*Linum usitatissimum*) (Wang et al., 2018). In this plant, *ABI3* produces three transcripts *LuABI3-1*, *LuABI3-2*, *LuABI3-3*. Protein containing all functional domains is produced from those transcripts in the translation process. However, only *LuABI3-1* and *LuABI3-2* generate functional proteins. The intron retention in the *LuABI3-3* pre-mRNA causes the generated protein not to exhibit biological activity; like the research under *SlABI3* isoforms in tomatoes and in flax transgenic plants overexpressing *LuABI3-1* or *LuABI3-2* affects the expression of seed-specific genes and thus regulates seed germination (Wang et al., 2018). The latest research on the three stages of seed development of alfalfa (*Medicago truncatula*) showed the presence of three *ABI3* splice isoforms (Lalanne et al., 2021). The first is the full-length MtABI3 protein named SPLICING FORM 1 (SF1), the transcript of which contains nine exons and all functional domains. The *SF2* isoform transcript is truncated, contains eight exons, and lacks the A1 activation domain. The pre-mRNA of the third isoform *SF3* contains six exons, the A1 domain, and truncated B1 and B3 domains but also lacks the B2 domain. The loss of functional domains in two isoforms caused both to have different cell functions. Moreover, each of these isoforms had a different, specific expression pattern during various phases of seed development and a different and specific pattern of expression depending on the type of tested tissue, i.e., embryo, endosperm, and seed coat. Analysis of the differential expressed genes (DEG) in hairy roots after the ectopic expression of individual splicing forms *SF1*, *SF2*, and *SF3* in the background of the *abi3* mutant revealed that only 41 genes are expressed commonly for all three *MtABI3* isoforms. However, 791 specific DEGs for *SF1*, 357 for *SF2*, and 177 for *SF3* were identified as involved in different biological processes. Specific DEGs in *SF1* were associated with photosynthesis, important storage proteins, the photosynthetic PSII complex, and the development of important storage proteins during the seed development process. In the case of *SF2*, the identified DEGs were associated with the plasma membrane intrinsic protein (PIP) and the degradation of fatty acids and the cell wall. On the other hand, the processes involving differential expressed genes specific to the third *SF3* isoform concerned the modification of the cell wall and the activity of pectin methyltransferases. Moreover, alternative splicing of *ABSCISIC ACID INSENSITIVE3/VIVIPAROUS1* (*ABI3/VP1*) homologs has also been reported in monocots such as wheat (*Triticum aestivum*) and rice (*Oryza sativa*) (McKibbin et al., 2002; Wilkinson et al., 2005; Fan et al., 2007). In both species, multiple *ABI3* isoforms have been identified that were generated from multiple different AS events, which usually resulted in an open reading frame (ORF) shift and generated a stop codon. Non-functional shortened proteins from aberrantly spliced transcripts are created, which is usually associated with lower seed quality. As a result, the seeds of these crops show reduced primary dormancy as increased pre-harvest sprouting (PHS).

In Arabidopsis, it is known that ABI3 directly interacts with transcription factor ABI5 *via* the B1 domain and promotes transcription of its target gene by binding to ABRE elements in the DNA (Nakamura et al., 2001). The *ABI5* homolog of Arabidopsis in rice *OsABI5* is alternatively spliced at the seed germination stage generating two variants of transcripts, the *OsABI5-1* and *OsABI5-2* (Zou et al., 2007). As a result of the exon skipping the event in the pre-mRNA *OsABI5*, the translated proteins differ in length in 10 amino acids localized after the bZIP domain. The *OsABI5-2* is an elongated protein version; AS changes the binding activity of *OsABI5* isoforms with ABI3. The ability to bind to ABI3 of both isoforms is preserved. However, the *OsABI5-2* isoform binding is much stronger. This influences the ABI5-ABI3 complex formation efficiency, suggesting different roles of those two *ABI5* isoforms in regulating the ABA signaling under changing environmental conditions (Zou et al., 2007). As previously mentioned, two alternatively spliced isoforms of *ABI5* have also been identified in Arabidopsis in recent studies of the *skip-1* mutant in response to ABA during seed germination (Zhang et al., 2022b).

The PHYTOCHROME INTERACTING FACTORS (PIFs) family consists of the eight proteins belonging to the helix-loop-helix (bHLH) TFs that modulate the response of plants to light and act as negative light regulators in the germination process (Leivar and Quail, 2011; Jeong and Choi, 2013; Leivar and Monte, 2014; Yang et al., 2020). PIF1 and PIF6 directly interact with Phytochrome B (PhyB) (Khanna et al., 2004; Narsai et al., 2017). It was revealed that in the presence of light, phytochromes, which are far-red and red-light receptors, are activated and bind to the transcription factor PHYTOCHROME INTERACTING FACTOR 1 (PIF1). This leads to the degradation of PIF1 in ubiquitin and the 26S proteasome pathway and, as a result, enables the seed to germinate (Oh et al., 2004; Shen et al., 2005). In contrast, in the absence of light, the PIF1 protein promotes GA degradation and inhibits the expression of genes from the GA biosynthetic pathway. At the same time, it increases the levels of ABA in seeds by promoting the expression of ABA biosynthesis genes and reducing the expression of genes involved in ABA catabolism (Finkelstein et al., 2008; Oh et al., 2009; Gabriele et al., 2010). In addition to affecting the metabolism of ABA and GA, PIF1 also directly binds to the *ABI3* and *ABI5* genes from the ABA signal transduction pathway and *GA INSENSITIVE DWARF1A* (*GID1A*) and *REPRESSOR OF GA1-3 1* (*RGA1*) genes from the GA signaling pathway (Oh et al., 2007; Oh et al., 2009; Kim et al., 2016a). PIF1 connects the light pathway with the ABA signaling pathway and binds to *ABI3* to activate the expression of the negative seed germination regulator SOMNUS (SOM) to inhibit germination of the seeds (Dong et al., 2008; Kim et al., 2008; Park et al., 2011). Regarding the PIF6 transcription factor, it has been shown that it undergoes AS during seed germination. The expression of PIF6 transcripts is particularly observed in dry seeds, and at the initial stage of germination, there is a strong reduction in its expression level. This expression pattern of PIF6 is contrasted with the PIF1, whose transcripts accumulate only at the imbibition stage (Penfield et al., 2010). As a result of the third exon skipping the event, two *PIF6* transcripts are generated, encoding two different proteins, PIF6-α and PIF6-β. The PIF6-α variant is a full-length protein, while the PIF6-β isoform is truncated and lost the helix-loop-helix bHLH domain essential for binding this transcription factor with DNA. Only seeds overexpressing PIF6-β germinate more intensively compared to the germination level of WT, while PIF6-α overexpression does not affect the germination ratio. This shows that AS caused those two *PIF6* isoforms to affect the seed germination rate differently. It indicates the antagonistic role of *PIF6-α* and *PIF6-β* in the germination process. It is worth mentioning that those two isoforms of *PIF6* do not always act differently. Overexpression of *PIF6-α* or *PIF6-β* inhibited the elongation of the seedling hypocotyl in R-light, which suggests a similar functioning of these isoforms in seedling development (Penfield et al., 2010). It was revealed that AS pre-mRNA *PIF6* is driven by the polypyrimidine tract-binding proteins (PTB), which are the component of the splicosome machinery. PTBs belong to heterogeneous nuclear ribonucleoproteins I (hnRNP I) and act as splicing suppressors (Sawicka et al., 2008; Dai et al., 2022). Arabidopsis genome has three *PTB* genes (*PTB1*, *PTB2*, and *PTB3*) and all of them undergo alternate splicing to generate two transcripts *SPLICING VARIANT I* (*SPI*) and *SPLICING VARIANT II* (*SPII)* (Stauffer et al., 2010). The pre-mRNA of *SPI* is formed into a protein with the correct length, while the *SPII* has PTC in its nucleotide sequence, which directs this transcript to the NMD pathway. This example shows the role of AS coupling with the NMD mechanism in regulating the level of functional proteins. PTB variants can mutually regulate their splicing but also show the autoregulation ability to balance AS events. Studies in Arabidopsis have shown that AS of *PIF6* is regulated only by PTB1 and PTB2 (Rühl et al., 2012). An opposite pattern of accumulation of *PIF6* splice isoforms was observed in mutants overexpressing or with knockdown of *PTB1* or *PTB2*. In the case of mutants with *PTB1* or *PTB2* overexpression and simultaneous overexpression of both *PTB*s, it led to the accumulation of the correct *PIF6-α* isoform. In contrast, the knockdown of single *PTB1* or *PTB2* or both *PTB*s promoted the truncated *PIF6-β* isoform. This contrary accumulation pattern of alternatively spliced *PIF6* isoforms has been associated with the altered germination ability of *PTB* mutants in the presence of ABA. Mutants overexpressing *PTB1* increased the levels of *PIF6-α* and delayed seed germination, suggesting that this protein acts as a negative germination regulator in the presence of ABA. In addition, it was confirmed by the observation that the accumulation of non-functional PIF6-β protein in a double mutant with switched off both *PTB1* and *PTB2* genes increased the seed germination potential in response to ABA. The results of these studies indicate the role of *PTB*s in the AS of *PIF6*. Also, other studies in germinating Arabidopsis seeds identified AS of *PIF6*, in which four differently expressed *PIF6* transcripts were detected (Narsai et al., 2017). Moreover, PhyB, which interacts with PIF6, also undergoes AS (Khanna et al., 2004; Narsai et al., 2017). Two *PhyB* transcripts have been identified that display isoform switch, which suggests that these isoforms may be important at different stages of seed germination. The expression of the first isoform is high in dry seeds and gradually decreases during stratification, while a gradual, high increase in expression is again observed upon exposure to light. In contrast, the expression of the second isoform is the highest during stratification (Narsai et al., 2017). PhyB interacts with the splicing factor DNA-DAMAGE REPAIR/TOLERATION PROTEIN111/SPLICING FACTOR FOR PHYTOCHROME SIGNALING (DRT111/SFPS), which plays a role in photomorphogenesis (Xin et al., 2017). It shows that DRT111 is a hub connecting light and ABA signaling to regulate seed germination (Punzo et al., 2020). The *drt111* mutant was observed to have altered expression of genes from the ABA signaling pathway, including ABI3 and the light signaling pathway e.g. *PIF1* and *PIF6*. Analysis of *drt111-2*/*sua-2* double mutants and *drt111-2/sua-2/abi3-5* triple mutants showed an interaction between *DRT111* and *SUA*. This indicates that DRT111 controls *ABI3* splicing *via* SUA. Variation in the *ABI3* AS isoforms was observed in the single *drt111-2* mutant, where only the non-functional *ABI3-β* isoform was increased in dry seeds. Interestingly, at the imbibition stage, both *ABI3-α* and *ABI3-β* pre-mRNA amounts increased, but ABI3-β levels rise four times more than WT.

## A key regulator of seed dormancy, DOG1 undergoes alternative splicing and interferes with ABA signaling components

Interestingly, the main dormancy regulator DELAY OF GERMINATION 1 (DOG1) has been shown to undergo AS, and recent studies have revealed that DOG1 also interacts with the ABA signaling components (Nakabayashi et al., 2015; Dekkers et al., 2016; Née et al., 2017). As a result of the alternative 3′ and 5′ splice sites selection AS events, five different *DOG1* transcripts are generated: *DOG1-α*, *DOG1-β*, *DOG1-γ*, *DOG1-δ*, and *DOG1-ϵ*. These transcripts are translated into three types of proteins because the same protein is produced from the *DOG1-β*, *DOG1-γ*, and *DOG1-ϵ* transcripts (Bentsink et al., 2006; Nakabayashi et al., 2015). Although these proteins vary in length, they are all biologically functional. The role of these splice variants of *DOG1* is unknown. However, current studies suggest they play a synergistic role in seed dormancy (Nakabayashi et al., 2015). Analysis of the phenotype of mutants with overexpression of the individual single *DOG1* isoform in the *dog1* mutant background showed that the seeds do not accumulate the DOG1 protein, resulting in no dormant seeds. However, deep dormancy is observed when the mutant overexpresses at least two *DOG1* isoforms. This phenotype has been associated with the accumulation of the DOG1 protein, which increases with the number of overexpressed isoforms. This shows that at least two *DOG1* isoforms are required to induce seed dormancy. The expression of DOG1 increases with seed development and reaches the highest levels till halfway through the seed maturation process and then decreases until the lowest level in the dry seeds. The rise in the expression of *DOG1* during seed maturation is influenced by low temperature and ABA treatment (Chiang et al., 2011; Kendall et al., 2011). The drop in temperature causes a higher amount of DOG1 protein in the cells, which promotes seed dormancy (Zaretskaya et al., 2022). Analysis of the *dog1-1* mutant revealed its non-dormant phenotype, which is similar to ABA-deficient and signaling mutants. The *dog1-2 cyp707a2-1* double mutant exhibited reduced dormancy compared to the ABA catabolism single mutant *cyp707a2*. The high ABA levels in the *dog1-2 cyp707a2-1* did not lead to seed dormancy (Nakabayashi et al., 2012). However, high levels of *DOG1* in the non-dormant ABA-deficient mutant *aba1* cannot inhibit the germination of the seeds (Bentsink et al., 2006). Interestingly, recent reports indicate that DOG1 enhances ABA signaling through the interaction and inhibition of PP2C ABA HIPERSENSITIVE GERMINATION1 (AHG1) and ABA HYPERSENSITIVE GERMINATION 3 (AHG3) phosphatases characteristic for seed development and germination (Yoshida et al., 2006; Nishimura et al., 2007; Née et al., 2017; Nishimura et al., 2018). Studies using mutants in the *AHG1* and *AHG3*, and *DOG1* genes have shown that the single *ahg1* and *ahg3* mutants are significantly less dormant than the *ahg1/ahg3* double mutant. On the other hand, double mutants *dog1/ahg1* or *dog1/ahg3* can still germinate, and only the loss of the function of all three genes causes seed dormancy. This indicates that AGH1 and AHG3 phosphatases may partially take over their functions and that they are downstream from DOG1 (Née et al., 2017; Nishimura et al., 2018). *AHG3* is regulated in the ABA signaling pathway dependent on PYRABACTIN RESISTANCE/PYR-LIKE PROTEIN/ABA RECEPTOR REGULATORY COMPONENTS (PYR/PYL RCAR) receptors and a separate pathway regulated by DOG1. In contrast, AHG1 phosphatase is regulated exclusively by DOG1).

## Regulation of ABA-related changes in alternative splicing by the spliceosomal machinery during seed germination

Interestingly, it has been shown that spliceosomal components are not only involved in the splicing mechanism but also undergo AS. Serine/arginine-rich (SR) and SR-like proteins belong to the conserved family of pre-mRNA splicing factors. The SR proteins influence the choice of the splicing site and regulate spliceosome assembly by binding to the cis-regulatory sequences in the pre-mRNA (Zahler et al., 1992; Reddy, 2007). In their structure, the SR proteins have one or two RRM motifs at the N-terminus responsible for RNA recognition and an arginine/serine-rich RS domain at the C-terminus allowing for the interaction with other proteins (Wu and Maniatis, 1993; Manley and Tacke, 1996; Graveley et al., 2001; Reddy, 2004; Day et al., 2012). The SR45 protein is an SR-like protein with an additional RS domain located at the N-terminus (Tanabe et al., 2007). In Arabidopsis, *SR45* is spliced at various stages of plant development or in response to external stresses (Ali et al., 2007; Zhang et al., 2014; Filichkin et al., 2015). AS for *SR45*, using alternative 3′ splice sites leads to the production of two isoforms. AS variant *SR45.1* is a full-length transcript, while *SR45.2* is a truncated version. These isoforms encode very similar proteins differing only in eight amino acids, which are only present in the *SR45.1* isoform. Despite this, both isoforms are functional, but surprisingly, each has different functions. The first isoform, *SR45.1*, plays a primary function in flower petal development, while *SR45.2* is in root growth (Ali et al., 2007; Zhang and Mount, 2009; Zhang et al., 2014). Moreover, SR45 has been shown to act in the ABA pathway mediated by glucose (Glc) (Carvalho et al., 2010). Glc induces the expression of ABA biosynthesis and signaling genes, which increases endogenous ABA levels and influences ABA signal transduction (Dekkers et al., 2008; Huang et al., 2016). Such a link between Glc-ABA was observed in the *sr45* mutant, which is hypersensitive to glucose and ABA at the seedling stage (Carvalho et al., 2010). The analysis of the *ABI3* and *ABI5* transcripts levels in the *sr45* mutant after treatment with ABA and glucose showed that these transcripts increased significantly compared to the wild type only after glucose treatment. This indicates that the SR45 protein has a role in the negative regulation of *ABI3* and *ABI5* transcription after glucose treatment. In contrast, reversing the hypersensitivity to Glc of mutant *sr45* in complemented lines with *SR45.1* and *SR45.2* reveals that these isoforms have the same function (Carvalho et al., 2010). Other research studies have shown that the regulatory role of SR45 in the pathway linking sugar signaling with ABA is to control the level of SNF1-RELATED PROTEIN KINASE 1 (SnRK1) in the cell by modulating the AS of INOSITOL POLYPHOSPHATE 5-PHOSPHATASE (5PTase13), which is involved in the proteasomal degradation of SnRK1 (Ananieva et al., 2008; Rodrigues et al., 2013). Analysis of the Arabidopsis seedling transcriptome identified over 4,000 RNAs that directly or indirectly bind to SR45 (Xing et al., 2015). The Gene Ontology (GO) of SR45-related RNA enabled it to indicate the main biological processes in which they are engaged and, through this, confirmed the role of SR45 in processes such as flowering, various aspects of plant development, and RNA splicing. Importantly, the highly enriched term GO responded to abiotic stresses and ABA. This showed that SR45 is involved in the ABA signaling pathway. Moreover, the *SR45* knockout affects genes’ transcription and the AS from the ABA signaling pathway (Xing et al., 2015). In the case of the *HAB1* gene in the *sr45* mutant, the transcription of this gene was reduced, but the accumulation of the AS spliced variant *HAB1.2* with IR was promoted in the control conditions and in the presence of ABA (Xing et al., 2015). In Arabidopsis, AS isoforms of *SR45* also function in response to salt stress (Albaqami et al., 2019). Knock-out of the *SR45* resulted in increased salt stress sensitivity at different plant life cycle moments, including seed germination. In the presence of salt stress, *sr45* mutant genes related to the *SALT OVERLY SENSITIVE* (*SOS*) pathway and ABA signaling were differentially expressed and/or exhibited changed splicing patterns. Interestingly, only the *SR45.1* long isoform reversed the salt-sensitive phenotype of the *sr45* mutant as well as altered the expression of these genes and splicing patterns. The obtained results indicate that SR45 can positively regulate the response to salt stress in Arabidopsis; in this response, only the longer isoform *SR45.1* is involved (Albaqami et al., 2019). SR45a is another splicing regulator that also undergoes AS and, together with the smaller subunit CAP-BINDING PROTEIN 20 (CBP20) of the CAP-BINDING COMPLEX (CBC), cooperates in the salt stress response (Li et al., 2021). SR45a, similar to SR45, has two RS domains, and because of AS, due to the loss of the C-terminal domain, two isoforms with altered functions are created (Tanabe et al., 2009; Li et al., 2021). SR45a-1a is a full-length functional protein variant, whereas the isoform SR45a-1b is truncated and incapable of interacting with other splicosome elements. However, SR45a-1b plays an important regulatory role. Both SR45a isoforms have the RS domain located at the N-terminal region, so both can interact with the CBP20 protein. In response to salt stress, the complex of SR45a-1a with CBP20 is created, and the binding of those two proteins is enhanced by SR45a-1b. This formed complex regulates the gene expression of the salt-responsive genes and modulates their AS. At the germination stage, the loss of function of *SR45a* confers tolerance to salinity. At the same time, overexpression of the *SR45a-1a* or *SR45a-1b* isoform increased the salinity sensitivity, suggesting a negative regulation of salt stress by the *SR45a* spliced variants (Li et al., 2021). The latest research also indicates the functionality of *SR45* isoforms in seed germination in Arabidopsis. The study of the Arabidopsis transcriptome dynamics during seed germination identified 620 genes that showed isoform variation. Moreover, 612 genes were differentially expressed, including *SR45* (Narsai et al., 2017).

## Transcriptome analyses reveal the involvement of alternative splicing in seeds

Although the amount of RNA-seq research on AS in plants is constantly growing, more data about seeds and the germination process still needs to be collected, especially in monocots. An RNA-seq study of germinating barley (*Hordeum vulgare*) embryos from four varieties identified 2,200 and 3,900 AS transcripts 24 and 48 h after imbibition (Zhang et al., 2016). The alternative 3′ splice site was the most common AS event, which amounted to 34% and 45% of all AS events. This observation was interesting because the most frequent event in plants is intron retention (Reddy et al., 2012). This research showed that AS process changes over time in germinating seeds. The number of detected transcripts after 24 h and 48 h was similar but not identical. Half of the transcripts present in the embryos after 24 h were no longer detectable at 48 h, but other new transcripts were produced at this time. Evaluating the biological functions of these identified AS genes showed their possible engagement in protein synthesis, energy and metabolism of carbon, and post-transcriptional regulation. Moreover, among AS genes, genes acting in the signaling pathways of various phytohormones have been identified. At the 24 and 48 h of germination, splicing variants of genes from the auxin, cytokinin, and the ABA signaling pathway, such as *AUXIN-RESPONSIVE PROTEIN IAA* (*AUS/IAA*) [alternative 3′ splice site (A3S)], *A-TYPE RESPONSE REGULATOR* (*A-ARR*) (IR), or *SnRK2* (A3S, IR), was detected. However, the A3S alternatively spliced variant of the *A-ARR* gene was present only for 24 h and the IR isoform of the *AUX/IAA* gene after 48 h. Exceptionally high levels of coexpressed genes had auxin efflux carrier, auxin-responsive AUX/IAA proteins, and SnRK2 from the ABA signaling pathway. This indicates that this seed germination stage involves phytohormones such as auxin and ABA. Therefore, the newly-created splice variants may differently modulate various signaling pathways and biological processes and thus affect seed germination. AS was also reported in rice seeds in response to stress conditions (Chen et al., 2019). In low oxygen conditions, 1,741 differentially expressed AS were identified. Interestingly, only 5% of AS genes were as well differentially expressed. That result reveals the independent transcription role of alternative splicing in gene regulation. A similar relation was found in the study of the Arabidopsis transcriptome, where only 6% of the identified genes that underwent AS were also differentially expressed (Srinivasan et al., 2016). In 14 and 20 days after pollination, 4,723 and 4,494 AS genes were identified, respectively. Those genes were mainly involved in processes related to mRNA catabolism. In total, 8,927 AS events were detected, of which 88% were newly identified in Arabidopsis. Among them was found a new alternative 3′ splice site (A3P) event of the *FUSCA3* (*FUS3*) gene which codes for a B3 domain transcription factor that acts as a regulator of seed development (Gazzarrini et al., 2004; Tsai and Gazzarrini, 2012). From this AS variant a truncated protein was created due to reading frame shift and formation of a premature stop codon within the B3 domain. This may influence FUS3 functionality and change its DNA binding activity. Although the function of this splice isoform is not determined, it is worth noting that the A3P *FUS3* was expressed explicitly at 20 DAP, in contrast to the full-length isoform which was downregulated. The high frequency of the AS process in seeds was also confirmed by RNA-seq studies in dicots (Aghamirzaie et al., 2013; Ruan et al., 2018; Feng et al., 2019; Liu et al., 2022). In the case of peanut (*Arachis hypogaea*) seeds, 30 days after flowering, 20,213 genes underwent AS, which is 49.69% of all expressed genes. In contrast, on the 50th day after flowering, 19,534 AS genes were identified, corresponding to 48.30% of all expressed genes. 92,483 and 85,562 AS events were detected at these two-time points, respectively. It is worth noting that in the seeds was the most significant number of AS isoforms compared to the root and leaf tissue. It was observed that a considerable percentage of genes that underwent AS was involved in the metabolism of fatty acids (approx. 60%), which is an important process for the development of oil seeds. This suggests that alternative splicing may be a crucial mechanism regulating this process. Also, the new splice variants of *Aradu.5N10F*, *Araip.84LR8*, and *Araip.92Q2X* genes encoding fatty acid desaturase (FAD) were detected. These isoforms were generated as a result of a novel exon presence. However, in the case of the *Araip.92Q2X*, an additional isoform was also produced which, except for an extra exon, had as well a 3′ alternative exon end (3′-AE). All those newly detected AS events did not disrupt the translation process and resulted in the formation of correct proteins (Ruan et al., 2018). In the soybean (Glycine max) case, 217,371 different AS transcript isoforms from 47,331 AS genes were identified at the stage of embryogenesis (Aghamirzaie et al., 2013). Moreover, RNA-seq studies of germinating seeds showed high AS process dynamics. In cotton (*Gossypium australe*), the greatest differences in AS were observed in comparison of the 5th and 30th hour of germination (Feng et al., 2019). Similarly, RNA-seq studies in Arabidopsis showed variable accumulation of AS isoforms at different phases of seed development and in various tissues, with the most significant changes when comparing seeds after imbibition with dry seeds (Narsai et al., 2017). The AS pattern was recently investigated in seeds of high and low secondary dormancy varieties of oilseed rape (*Brassica napus*) after polyethylene glycol 6000 (PEG6000) treatment to induce secondary dormancy. After treatment with PEG, 5,136 genes that underwent AS were identified, accounting for 8% of all expressed genes. From these genes, 11,408 splicing variants have been detected, of which over 50% were generated due to intron retention. Among the differentially alternatively spliced genes, a large group consisted of genes encoding spliceosome components, e.g., small nuclear ribonucleoprotein molecules (snRNPs), serine/arginine-rich proteins (SR) whose function in Arabidopsis has been confirmed in the process of embryo development (*JANUS*), seed germination (*RZ-1C*, *RBM25*) or response to stress conditions (*SCL30A*, *U1-70K*, *SR45A*). Moreover, within the differentially alternatively spliced genes, 342 genes played a role in the secondary dormancy, with only seven of them also significantly expressed. Interestingly, the expression of genes that underwent AS and were also differentially expressed was more than three times higher than the rest of the differentially expressed genes, indicating a correlation between changes in the AS process with changes at the transcription level. However, comparing the number of differentially alternatively spliced genes with differentially expressed genes showed that the number of unique genes is greater than the number of common genes and that suggests an independent action of AS process and transcription in seed dormancy of oilseed rape (Liu et al., 2022).

The RNA-seq data combined with proteomics analysis complements the information about the functions of generated splicing isoforms and may enrich the knowledge of the mechanisms regulating the seed germination process. This approach was used in the study of maize (*Zea mays*) seed germination in the presence of a high concentration of NaCl. It was noted that during germination, the reaction to the salt stress of the embryo and the endosperm is different. In the salt stress conditions, the embryo could germinate, whereas the germination of seeds with endosperm was inhibited. However, in the presence of ABA, germination was strongly reduced in both cases. These interesting observations suggest that, unlike the endosperm, the embryo is not responsible for the perception of the NaCl, but the signal transduction of the ABA is active. The transcriptomic and proteomics analyses showed significant differences in posttranscriptional events and translational regulation between the embryo and the endosperm. Comparable amounts of differentially expressed posttranscriptional events (DPTE) were detected in the embryo and the endosperm tissue, respectively – 14,570 events of 4,529 genes in the embryo and 15,023 events of 4,756 genes in the endosperm. These events included processes that generate different mRNA isoforms, such as the alternative start of transcription, alternative splicing, and alternative polyadenylation. In contrast, few differentially expressed genes were detected in both examined tissues, just 57 in the embryo and 148 in the endosperm. However, proteomics analysis showed the presence of 243 proteins with increased and 419 proteins with reduced expression in the embryo after NaCl treatment. In the case of endosperm, these amounts amounted to 389 and 234, respectively. Interestingly, among the proteins that were produced as a result of DPTE, 11 splicing factors were identified, and their expression at the protein level was differently regulated in the embryo and endosperm. Together, these studies indicate that posttranscriptional events, including AS, may be crucial for seed germination in response to salt stress and that the different expression patterns of splicing factors can be connected with the opposite reaction to NaCl of the embryo and endosperm in maize (Chen et al., 2021).

## Third-generation sequencing (TGS) in plant transcriptome studies

So far, genome-wide analysis of AS has been primarily based on high-throughput sequencing technology using the RNA-seq Illumina short-read approach. However, detecting various isoforms, repeated sequences, and transposable elements using Illumina is difficult. This is because it is impossible to distinguish different transcripts with identical exons, so reconstructing full-length alternative transcripts is demanding (Bernard et al., 2014). TGS like Pacific Biosciences (PacBio) and Oxford Nanopore Technologies (ONT) overcome these limitations due to the use of SMRT sequencing technology which allows the sequencing of single molecules in real time (Zhao et al., 2019). The long-read RNA sequencing technologies can sequence full-length transcripts and obtain long cDNA or RNA reads. This allows for identifying new isoforms of transcripts, protein-coding or non-protein–coding genes, and alternative polyadenylation sites. Long reads also remarkably improve the plants’ transcriptome annotation (Abdel-Ghany et al., 2016; Zhang et al., 2019; Cui et al., 2020). This together makes PacBio and ONT more often used to analyze plant transcriptomes. TGS has been recently applied in transcriptomic studies of many plant species such as maize (*Zea mays*) (Wang et al., 2016), rice (*Oryza sativa*) (Zhang et al., 2019), sorghum (*Sorghum bicolor*) (Abdel-Ghany et al., 2016), rapeseed (*Brassica napus*) (Yao et al., 2020), strawberry (*Fragaria vesca*) (Li et al., 2018), bamboo (*Phyllostachys edulis*) (Wang et al., 2020), Arabidopsis (Li et al., 2016), and tea plant (*Camellia sinensis*) (Qiao et al., 2019). A maize multi-tissue analysis using PacBio revealed 111,151 transcripts, of which 57% represent a novel, also specific for the tissue isoforms. By discovering new genes, isoforms, and lncRNAs, this method more than doubled the number of alternative transcripts and updated the annotation of the maize genome (Wang et al., 2016). To increase assembly quality and reduce the error rate, hybrid sequencing combining NGS and TGS approaches is applied. In the transcriptome study by PacBio and Illumina of adzuki bean (*Vigna angularis*), two varieties during seed germination under drought stress, 2,457 gene loci, and 46,177 isoforms were newly discovered, and the quality of transcript was significantly improved (Zhu et al., 2020).

## Concluding remarks

Alternative splicing (AS) is a crucial co-transcriptional mechanism that regulates gene expression in response to internal cues and environmental signals. Advances in high-throughput sequencing (HTS) technology showed plants’ increasing frequency of AS events. However, there still needs to be more information regarding the factors that regulate AS and the effects of AS on mRNA levels and protein functioning. Considering the link between AS and seed germination, further studies are needed to uncover the complex regulatory networks of seed germination and reveal parts of splice variants. This opens another exciting area for research. The obtained knowledge can be used to regulate gene expression to develop crops with new and desirable functional traits.

## Author contributions

ES wrote the first draft of the manuscript and prepared the figures and table. AD-G contributed to the conception and design of the manuscript and revised it critically for important intellectual content. All authors contributed to the article and approved the submitted version.

## References

[B1] Abdel-Ghany S. E. Hamilton M. Jacobi J. L. Ngam P. Devitt N. Schilkey F.. (2016). A survey of the sorghum transcriptome using single-molecule long reads. Nat. Commun. 7, 11706. doi: 10.1038/ncomms11706 27339290 PMC4931028

[B2] Aghamirzaie D. Nabiyouni M. Fang Y. Klumas C. Heath L. S. Grene R.. (2013). Changes in RNA splicing in developing soybean (Glycine max) embryos. Biology 2 (4), 1311–1337. doi: 10.3390/biology2041311 24833227 PMC4009788

[B3] Albaqami M. Laluk K. Reddy A. S. N. (2019). The arabidopsis splicing regulator SR45 confers salt tolerance in a splice isoform-dependent manner. Plant Mol. Biol. 100 (4–5), 379–390. doi: 10.1007/s11103-019-00864-4 30968308

[B4] Ali G. S. Palusa S. G. Golovkin M. Prasad J. Manley J. L. Reddy A. S. (2007). Regulation of plant developmental processes by a novel splicing factor. PloS One 2, e471. doi: 10.1371/journal.pone.0000471 17534421 PMC1868597

[B5] Ananieva E. A. Gillaspy G. E. Ely A. Burnette R. N. Erickson F. L. (2008). Interaction of the WD40 domain of a myoinositol polyphosphate 5-phosphatase with SnRK1 links inositol, sugar, and stress signaling. Plant Physiol. 148 (4), 1868–1882. doi: 10.1104/pp.108.130575 18931139 PMC2593651

[B6] Barbazuk W. B. Fu Y. McGinnis K. M. (2008). Genome-wide analyses of alternative splicing in plants: Opportunities and challenges. Genome Res. 18 (9), 1382–1391. doi: 10.1101/gr.053678.106 18669480

[B7] Bartel D. P. (2004). MicroRNAs: genomics, biogenesis, mechanism, and function. Cell 116, 281–297. doi: 10.1016/S0092-8674(04)00045-5 14744438

[B8] Bentsink L. Jowett J. Hanhart C. J. Koornneef M. (2006). Cloning of DOG1, a quantitative trait locus controlling seed dormancy in arabidopsis. Proc. Natl. Acad. Sci. U.S.A. 103, 17042–17047. doi: 10.1073/pnas.0607877103 17065317 PMC1636575

[B9] Bernard E. Jacob L. Mairal J. Vert J. P. (2014). Efficient RNA isoform identification and quantification from RNA-seq data with network flows. Bioinformatics 30 (17), 2447–2455. doi: 10.1093/bioinformatics/btu317 24813214 PMC4147886

[B10] Bi Y. Deng Z. Ni W. Shrestha R. Savage D. Hartwig T.. (2021). Arabidopsis ACINUS is O-glycosylated and regulates transcription and alternative splicing of regulators of reproductive transitions. Nat. Commun. 12, 945. doi: 10.1038/s41467-021-20929-7 33574257 PMC7878923

[B11] Bielewicz D. Kalak M. Kalyna M. Windels D. Barta A. Vazquez F.. (2013). Introns of plant pri-miRNAs enhance miRNA biogenesis. EMBO Rep. 14 (7), 622–628. doi: 10.1038/embor.2013.62 23681439 PMC3701235

[B12] Bies-Etheve N. da Silva Conceicao A. Koornneef M. Léon-Kloosterziel K. Valon C. Delseny M. (1999). Importance of the B2 domain of the arabidopsis ABI3 protein for em and 2S albumin gene regulation. Plant Mol. Biol. 6, 1045–1054. doi: 10.1023/A:1006252512202 10527428

[B13] Bonnal S. Martínez C. Förch P. Bachi A. Wilm M. Valcárcel J. (2008). RBM5/Luca-15/H37 regulates fas alternative splice site pairing after exon definition. Mol. Cell 32 (1), 81–95. doi: 10.1016/j.molcel.2008.08.008 18851835

[B14] Carlson S. M. Soulette C. M. Yang Z. Elias J. E. Brooks A. N. Gozani O. (2017). RBM25 is a global splicing factor promoting inclusion of alternatively spliced exons and is itself regulated by lysine mono-methylation. J. Biol. Chem. 292 (32), 13381–13390. doi: 10.1074/jbc.M117.784371 28655759 PMC5555197

[B15] Carvalho R. F. Carvalho S. D. Duque P. (2010). The plant-specific SR45 protein negatively regulates glucose and ABA signaling during early seedling development in arabidopsis. Plant Physiol. 154 (2), 772–783. doi: 10.1104/pp.110.155523 20699397 PMC2949030

[B16] Chang N. Sun Q. Hu J. An C. Gao H. (2017). Large Introns of 5 to 10 kilo base pairs can be spliced out in arabidopsis. Genes 8, 200. doi: 10.3390/genes8080200 28800125 PMC5575664

[B17] Chaudhary S. Jabre I. Reddy A. S. N. Staiger D. Syed N. H. (2019a). Perspective on alternative splicing and proteome complexity in plants. Trends Plant Sci. 24, 496–506. doi: 10.1016/j.tplants.2019.02.006 30852095

[B18] Chaudhary S. Khokhar W. Jabre I. Reddy A. S. N. Byrne L. J. Wilson C. M.. (2019b). Alternative splicing and protein diversity: Plants versus animals. Front. Plant Sci. 10. doi: 10.3389/fpls.2019.00708 PMC6581706 31244866

[B19] Chen M. Lu C. Sun P. Nie Y. Tian Y. Hu Q.. (2021). Comprehensive transcriptome and proteome analyses reveal a novel sodium chloride responsive gene network in maize seed tissues during germination. Plant Cell Environ 44, 88–101. doi: 10.1111/pce.13849 32677712

[B20] Chen M. X. Zhu F. Y. Wang F. Z. Ye N. H. Gao B. Chen X.. (2019). Alternative splicing and translation play important roles in hypoxic germination in rice. J. Exp. Bot. 70 (3), 885–895. doi: 10.1093/jxb/ery393 30535157 PMC6363088

[B21] Cheng C. Wang Z. Yuan B. Li X. (2017). RBM25 mediates abiotic responses in plants. Front. Plant Sci. 8. doi: 10.3389/fpls.2017.00292 PMC5344909 28344583

[B22] Chiang G. C. K. Bartsch M. Barua D. Nakabayashi K. Debieu M. Kronholm I.. (2011). DOG1 expression is predicted by the seed-maturation environment and contributes to geographical variation in germination in arabidopsis thaliana. Mol. Ecol. 20 (16), 3336–3349. doi: 10.1111/j.1365-294X.2011.05181.x 21740475

[B23] Cui J. Shen N. Lu Z. Xu G. Wang Y. Jin B. (2020). Analysis and comprehensive comparison of PacBio and nanopore-based RNA sequencing of the arabidopsis transcriptome. Plant Methods 16, 85. doi: 10.1186/s13007-020-00629-x 32536962 PMC7291481

[B24] Cui Z. Tong A. Huo Y. Yan Z. Yang W. Yang X.. (2017). SKIP controls flowering time *via* the alternative splicing of SEF pre-mRNA in arabidopsis. BMC Biol. 15, 80. doi: 10.1186/s12915-017-0422-2 28893254 PMC5594616

[B25] Dai S. Wang C. Zhang C. Feng L. Zhang W. Zhou X.. (2022). PTB: Not just a polypyrimidine tract-binding protein. J. Cell. Physiol. 237, 2357–2373. doi: 10.1002/jcp.30716 35288937

[B26] Daszkowska-Golec A. Skubacz A. Marzec M. Slota M. Kurowska M. Gajecka M.. (2017). Mutation in HvCBP20 (Cap binding protein 20) adapts barley to drought stress at phenotypic and transcriptomic levels. Front. Plant Sci. 8. doi: 10.3389/fpls.2017.00942 PMC5454077 28626467

[B27] Daszkowska-Golec A. Wojnar W. Rosikiewicz M. Szarejko I. Maluszynski M. Szweykowska-Kulinska Z.. (2013). Arabidopsis suppressor mutant of abh1 shows a new face of the already known players: ABH1 (CBP80) and ABI4-in response to ABA and abiotic stresses during seed germination. Plant Mol. Biol. 81 (1–2), 189–209. doi: 10.1007/s11103-012-9991-1 23196831 PMC3527740

[B28] Day I. S. Golovkin M. Palusa S. G. Link A. Ali G. S. Thomas J.. (2012). Interactions of SR45, an SR-like protein, with spliceosomal proteins and an intronic sequence: Insights into regulated splicing. Plant J. 71 (6), 936–947. doi: 10.1111/j.1365-313X.2012.05042.x 22563826

[B29] Dekkers B. J. W. He H. Hanson J. Willems L. A. J. Jamar D. C. L. Cueff G.. (2016). The arabidopsis delay of germination 1 gene affects abscisic acid insensitive 5 (ABI5) expression and genetically interacts with ABI3 during arabidopsis seed development. Plant J. 85 (4), 451–465. doi: 10.1111/tpj.13118 26729600

[B30] Dekkers B. J. Schuurmans J. A. Smeekens S. C.. (2008). Interaction between sugar and abscisic acid signalling during early seedling development in arabidopsis. Plant Mol. Biol. 67, 151–167. doi: 10.1007/s11103-008-9308-6 18278579 PMC2295253

[B31] Dong H. K. Yamaguchi S. Lim S. Oh E. Park J. Hanada A.. (2008). SOMNUS, a CCCH-type zinc finger protein in arabidopsis, negatively regulates light-dependent seed germination downstream of PIL5. Plant Cell 20 (5), 1260–1277. doi: 10.1105/tpc.108.058859 18487351 PMC2438461

[B32] Drechsel G. Kahles A. Kesarwani A. K. Stauffer E. Behr J. Drewe P.. (2013). Nonsense-mediated decay of alternative precursor mRNA splicing variants is a major determinant of the arabidopsis steady state transcriptome. Plant Cell 25 (10), 3726–3742. doi: 10.1105/tpc.113.115485 24163313 PMC3877825

[B33] Ezcurra I. Wycliffe P. Nehlin L. Ellerström M. Rask L. (2000). Transactivation of the brassica napus napin promoter by ABI3 requires interaction of the conserved B2 and B3 domains of ABI3 with different cis-elements: B2 mediates activation through an ABRE, whereas B3 interacts with an RY/G-box. Plant J. 24 (1), 57–66. doi: 10.1046/j.1365-313X.2000.00857.x 11029704

[B34] Fan J. Niu X. Wang Y. Ren G. Zhuo T. Yang Y.. (2007). Short, direct repeats (SDRs)-mediated post-transcriptional processing of a transcription factor gene OsVP1 in rice (Oryza sativa). J. Exp. Bot. 58 (13), 3811–3817. doi: 10.1093/jxb/erm231 18057047

[B35] Feng J. Li J. Gao Z. Lu Y. Yu J. Zheng Q.. (2015). SKIP confers osmotic tolerance during salt stress by controlling alternative gene splicing in arabidopsis. Mol. Plant 8 (7), 1038–1052. doi: 10.1016/j.molp.2015.01.011 25617718

[B36] Feng S. Xu M. Liu F. Cui C. Zhou B. (2019). Reconstruction of the full-length transcriptome atlas using PacBio iso-seq provides insight into the alternative splicing in gossypium australe. BMC Plant Biol. 19, 365. doi: 10.1186/s12870-019-1968-7 31426739 PMC6701088

[B37] Filichkin S. A. Cumbie J. S. Dharmawardhana P. Jaiswal P. Chang J. H. Palusa S. G.. (2015). Environmental stresses modulate abundance and timing of alternatively spliced circadian transcripts in arabidopsis. Mol. Plant 8 (2), 207–227. doi: 10.1016/j.molp.2014.10.011 25680774

[B38] Finch-Savage W. E. Footitt S. (2017). Seed dormancy cycling and the regulation of dormancy mechanisms to time germination in variable field environments. J. Exp. Bot. 68, 843–856. doi: 10.1093/jxb/erw477 28391330

[B39] Finkelstein R. Reeves W. Ariizumi T. Steber C. (2008). Molecular aspects of seed dormancy. Annu. Rev. Plant Biol. 59, 387–415. doi: 10.1146/annurev.arplant.59.032607.092740 18257711

[B40] Fujii H. Chinnusamy V. Rodrigues A. Rubio S. Antoni R. Park S.-Y.. (2009). *In vitro* reconstitution of an abscisic acid signaling pathway. Nature 462, 660–664. doi: 10.1038/nature08599 19924127 PMC2803041

[B41] Gabriele S. Rizza A. Martone J. Circelli P. Costantino P. Vittorioso P. (2010). The dof protein DAG1 mediates PIL5 activity on seed germination by negatively regulating GA biosynthetic gene AtGA3ox1. Plant J. 61 (2), 312–323. doi: 10.1111/j.1365-313X.2009.04055.x 19874540

[B42] Gagete A. P. Riera M. Franco L. Rodrigo M. I. (2009). Functional analysis of the isoforms of an ABI3-like factor of pisum sativum generated by alternative splicing. J. Exp. Bot. 60 (6), 1703–1714. doi: 10.1093/jxb/erp038 19261920 PMC2671620

[B43] Gao Y. Liu J. Zhang Z. Sun X. Zhang N. Fan J.. (2013). Functional characterization of two alternatively spliced transcripts of tomato ABSCISIC ACID INSENSITIVE3 (ABI3) gene. Plant Mol. Biol. 82 (1–2), 131–145. doi: 10.1007/s11103-013-0044-1 23504452

[B44] Gazzarrini S. Tsuchiya Y. Lumba S. Okamoto M. McCourt P. (2004). The transcription factor FUSCA3 controls developmental timing in arabidopsis through the hormones gibberellin and abscisic acid. Dev. Cell 7, 373–385. doi: 10.1016/j.devcel.2004.06.017 15363412

[B45] Gehring N. H. Roignant J. Y. (2021). Anything but ordinary - emerging splicing mechanisms in eukaryotic gene regulation. Trends Genet. 37, 355–372. doi: 10.1016/j.tig.2020.10.008 33203572

[B46] Graveley B. R. Hertel K. J. Maniatis T. O. M. (2001). The role of U2AF35 and U2AF65 in enhancer-dependent splicing. RNA 7 (6), 806–818. doi: 10.1017/S1355838201010317 11421359 PMC1370132

[B47] Gurnari C. Pagliuca S. Visconte V. (2021). Alternative splicing in myeloid malignancies. Biomedicines 9, 1844. doi: 10.3390/biomedicines9121844 34944660 PMC8698609

[B48] Hobo T. Kowyama Y. Hattori T. Phinney B. O. (1999). A bZIP factor, TRAB1, interacts with VP1 and mediates abscisic acid-induced transcription. Proc. Natl. Acad. Sci. U.S.A. 96, 15348–15353. doi: 10.1073/pnas.96.26.15348 10611387 PMC24822

[B49] Hong Y. Yao J. Shi H. Chen Y. Zhu J.-K. Wang Z. (2021). The arabidopsis spliceosomal protein SmEb modulates ABA responses by maintaining proper alternative splicing of HAB1. Stress Biol. 1, 1–8. doi: 10.1007/s44154-021-00006-1 PMC10441929 37676319

[B50] Huang H. Xie S. Xiao Q. Wei B. Zheng L. Wang Y.. (2016). Sucrose and ABA regulate starch biosynthesis in maize through a novel transcription factor, ZmEREB156. Sci. Rep. 10, 27590. doi: 10.1038/srep27590 PMC4901336 27282997

[B51] Hugouvieux V. Kwak J. M. Schroeder J. I. (2001). An mRNA cap-binding protein, ABH1, modulates early abscisic acid signal transduction in arabidopsis. Cell 106, 477–487. doi: 10.1016/S0092-8674(01)00460-3 11525733

[B52] Hugouvieux V. Murata Y. Young J. J. Kwak J. M. Mackesy D. Z. Schroeder J. I. (2002). Localization, ion channel regulation, and genetic interactions during abscisic acid signaling of the nuclear mRNA cap-binding protein, ABH1. Plant Physiol. 130 (3), 1276–1287. doi: 10.1104/pp.009480 12427994 PMC166648

[B53] Jäger K. Fábián A. Tompa G. Deák C. Höhn M. Olmedilla A.. (2011). New phenotypes of the drought-tolerant cbp20 arabidopsis thaliana mutant have changed epidermal morphology. Plant Biol. 13 (1), 78–84. doi: 10.1111/j.1438-8677.2010.00343.x 21143728

[B54] Jeong J. Choi G. (2013). Phytochrome-interacting factors have both shared and distinct biological roles. Mol. Cells 35, 371–380. doi: 10.1007/s10059-013-0135-5 23708772 PMC3887866

[B55] Kalyna M. Simpson C. G. Syed N. H. Lewandowska D. Marquez Y. Kusenda B.. (2012). Alternative splicing and nonsense-mediated decay modulate expression of important regulatory genes in arabidopsis. Nucleic Acids Res. 40 (6), 2454–2469. doi: 10.1093/nar/gkr932 22127866 PMC3315328

[B56] Kathare P. K. Huq E. (2021). Light-regulated pre-mRNA splicing in plants. Curr. Opin. Plant Biol. 63, 1–13. doi: 10.1016/j.pbi.2021.102037 PMC8487434 33823333

[B57] Kendall S. L. Hellwege A. Marriot P. Whalley C. Graham I. A. Penfield S. (2011). Induction of dormancy in arabidopsis summer annuals requires parallel regulation of DOG1 and hormone metabolism by low temperature and CBF transcription factors. Plant Cell 23 (7), 2568–2580. doi: 10.1105/tpc.111.087643 21803937 PMC3226211

[B58] Khanna R. Huq E. Kikis E. A. Al-Sady B. Lanzatella C. Quail P. H. (2004). A novel molecular recognition motif necessary for targeting photoactivated phytochrome signaling to specific basic helix-loop-helix transcription factors. Plant Cell 16 (11), 3033–3044. doi: 10.1105/tpc.104.025643 15486100 PMC527196

[B59] Kim J. Kang H. Park J. Kim W. Yoo J. Lee N.. (2016). PIF1-interacting transcription factors and their binding sequence elements determine the *in vivo* targeting sites of PIF1. Plant Cell 28 (6), 1388–1405. doi: 10.1105/tpc.16.00125 27303023 PMC4944412

[B60] Kim D. H. Yamaguchi S. Lim S. Oh E. Park J. Hanada A.. (2008). SOMNUS, a CCCH-type zinc finger protein in arabidopsis, negatively regulates light-dependent seed germination downstream of PIL5. Plant Cell 20 (5), 1260–1277. doi: 10.1105/tpc.108.058859 18487351 PMC2438461

[B61] Kurup S. Jones H. D. Holdsworth M. J. (2000). Interactions of the developmental regulator ABI3 with proteins identified from developing arabidopsis seeds. Plant J. 21, 143–155. doi: 10.1046/j.1365-313x.2000.00663.x 10743655

[B62] Lalanne D. Malabarba J. Ly Vu J. Hundertmark M. Delahaie J. Leprince O.. (2021). Medicago abi3 splicing isoforms regulate the expression of different gene clusters to orchestrate seed maturation. Plants 10, 1710. doi: 10.3390/plants10081710 34451755 PMC8398556

[B63] Lara P. Oñate-Sánchez L. Abraham Z. Ferrándiz C. Díaz I. Carbonero P.. (2003). Synergistic activation of seed storage protein gene expression in arabidopsis by ABI3 and two bZIPs related to OPAQUE2. J. Biol. Chem. 278 (23), 21003–21011. doi: 10.1074/jbc.M210538200 12657652

[B64] Laubinger S. Sachsenberg T. Zeller G. Busch W. Lohmann J. U. Rascht G.. (2008). Dual roles of the nuclear cap-binding complex and SERRATE in pre-mRNA splicing and microRNA processing in arabidopsis thaliana. Proc. Natl. Acad. Sci. U.S.A. 105, 8795–8800. doi: 10.1073/pnas.0802493105 18550839 PMC2426964

[B65] Leivar P. Monte E. (2014). PIFs: Systems integrators in plant development. Plant cell 26, 56–78. doi: 10.1105/tpc.113.120857 24481072 PMC3963594

[B66] Leivar P. Quail P. H. (2011). PIFs: Pivotal components in a cellular signaling hub. Trends Plant Sci. 16 (1), 19–28. doi: 10.1016/j.tplants.2010.08.003 20833098 PMC3019249

[B67] Li Y. Guo Q. Liu P. Huang J. Zhang S. Yang G.. (2021). Dual roles of the serine/arginine-rich splicing factor SR45a in promoting and interacting with nuclear cap-binding complex to modulate the salt-stress response in arabidopsis. New Phytol. 230 (2), 641–655. doi: 10.1111/nph.17175 33421141

[B68] Li Y. Wei W. Feng J. Luo H. Pi M. Liu Z.. (2018). Genome re-annotation of the wild strawberry fragaria vesca using extensive illumina-and SMRT-based RNA-seq datasets. DNA Res. 25 (1), 61–70. doi: 10.1093/dnares/dsx038 29036429 PMC5824900

[B69] Li S. Yamada M. Han X. Ohler U. Benfey P. N. (2016). High-resolution expression map of the arabidopsis root reveals alternative splicing and lincRNA regulation. Dev. Cell 39 (4), 508–522. doi: 10.1016/j.devcel.2016.10.012 27840108 PMC5125536

[B70] Li M. Yu B. (2021). Recent advances in the regulation of plant miRNA biogenesis. RNA Biol. 18, 2087–2096. doi: 10.1080/15476286.2021.1899491 33666136 PMC8632083

[B71] Lin J. Zhu Z. (2021). Plant responses to high temperature: a view from pre-mRNA alternative splicing. Plant Mol. Biol. 105, 575–583. doi: 10.1007/s11103-021-01117-z 33550520

[B72] Liu L. Wu D. Gu Y. Liu G. Liu B. Mao F.. (2022). Comprehensive profiling of alternative splicing landscape during secondary dormancy in oilseed rape (Brassica napus l.). Mol. Breed. 42, 44. doi: 10.1007/s11032-022-01314-8 PMC10248609 37313517

[B73] Liu F. Zhang H. Ding L. Soppe W. J. Xiang Y. (2020). Reversal of rdo51, a homolog of rice seed dormancy4, interacts with bHLH57 and controls ABA biosynthesis and seed dormancy in *Arabidopsis* . Plant Cell 32, 1933–1948. doi: 10.1105/tpc.20.00026 32213638 PMC7268807

[B74] Liu X. Zhang H. Zhao Y. Feng Z. Li Q. Yang H. Q.. (2013). Auxin controls seed dormancy through stimulation of abscisic acid signaling by inducing ARF-mediated ABI3 activation in *Arabidopsis* . Proc. Natl. Acad. Sci. U.S.A. 110, 15485–15490. doi: 10.1046/j.1365-313X.2002.01430.x 23986496 PMC3780901

[B75] Llorca C. M. Potschin M. Zentgraf U. (2014). bZIPs and WRKYs: two large transcription factor families executing two different functional strategies. Front. Plant Sci. 5. doi: 10.3389/fpls.2014.00169 PMC4012195 24817872

[B76] Lopez-Molina L. Mongrand S. McLachlin D. T. Chait B. T. Chua N. H. (2002). ABI5 acts downstream of ABI3 to execute an ABA-dependent growth arrest during germination. Plant J. 32, 317–328. doi: 10.1046/j.1365-313X.2002.01430.x 12410810

[B77] McCarty D. R. Hattori T. Carson C. B. Vasil V. Lazar M. Vasil I. K. (1991). The Viviparous-1 developmental gene of maize encodes a novel transcriptional activator. Cell 66, 895–905. doi: 10.1016/0092-8674(91)90436-3 1889090

[B78] Manley J. L. Tacke R. (1996). SR proteins and splicing control. Genes Dev. 10 (13), 1569–1579. doi: 10.1101/gad.10.13.1569 8682289

[B79] Marquez Y. Brown J. W. S. Simpson C. Barta A. Kalyna M. (2012). Transcriptome survey reveals increased complexity of the alternative splicing landscape in arabidopsis. Genome Res. 22 (6), 1184–1195. doi: 10.1101/gr.134106.111 22391557 PMC3371709

[B80] McKibbin R. S. Wilkinson M. D. Bailey P. C. Flintham J. E. Andrew L. M. Lazzeri P. A.. (2002). Transcripts of vp-1 homeologues are misspliced in modern wheat and ancestral species. Proc. Natl. Acad. Sci. U.S.A. 99, 10203–10208. doi: 10.1073/pnas.152318599 12119408 PMC126648

[B81] Meyer K. Koester T. Staiger D. (2015). Pre-mRNA splicing in plants: *In vivo* functions of RNA-binding proteins implicated in the splicing process. Biomolecules 5, 1717–1740. doi: 10.3390/biom5031717 26213982 PMC4598772

[B82] Muhammad S. Xu X. Zhou W. Wu L. (2022). Alternative splicing: An efficient regulatory approach towards plant developmental plasticity. Wiley Interdiscip. Reviews: RNA 9, 1–26. doi: 10.1002/wrna.1758 35983878

[B83] Murachelli A. G. Ebert J. Basquin C. Le Hir H. Conti E. (2012). The structure of the ASAP core complex reveals the existence of a pinin-containing PSAP complex. Nat. Struct. Mol. Biol. 19 (4), 378–386. doi: 10.1038/nsmb.2242 22388736

[B84] Nakabayashi K. Bartsch M. Ding J. Soppe W. J. J. (2015). Seed dormancy in arabidopsis requires self-binding ability of DOG1 protein and the presence of multiple isoforms generated by alternative splicing. PloS Genet. 11, e1005737. doi: 10.1371/journal.pgen.1005737 26684465 PMC4686169

[B85] Nakabayashi K. Bartsch M. Xiang Y. Miatton E. Pellengahr S. Yano R.. (2012). The time required for dormancy release in arabidopsis is determined by DELAY OF GERMINATION1 protein levels in freshly harvested seeds. Plant Cell 24 (7), 2826–2838. doi: 10.1105/tpc.112.100214 22829147 PMC3426117

[B86] Nakamura S. Lynch T. J. Finkelstein R. R. (2001). Physical interactions between ABA response loci of arabidopsis. Plant J. 26 (6), 627–635. doi: 10.1046/j.1365-313X.2001.01069.x 11489176

[B87] Narsai R. Gouil Q. Secco D. Srivastava A. Karpievitch Y. V. Liew L. C.. (2017). Extensive transcriptomic and epigenomic remodelling occurs during arabidopsis thaliana germination. Genome Biol. 18, 172. doi: 10.1186/s13059-017-1302-3 28911330 PMC5599894

[B88] Nasif S. Contu L. Mühlemann O. (2017) Beyond quality control: The role of nonsense-mediated mRNA decay (NMD) in regulating gene expression. Available at: http://www.elsevier.com/open-access/userlicense/1.0/. 10.1016/j.semcdb.2017.08.053 28866327

[B89] Née G. Kramer K. Nakabayashi K. Yuan B. Xiang Y. Miatton E.. (2017). DELAY of GERMINATION1 requires PP2C phosphatases of the ABA signalling pathway to control seed dormancy. Nat. Commun. 8, 72. doi: 10.1038/s41467-017-00113-6 28706187 PMC5509711

[B90] Nilsen T. W. Graveley B. R. (2010). Expansion of the eukaryotic proteome by alternative splicing. Nature 463, 457–463. doi: 10.1038/nature08909 20110989 PMC3443858

[B91] Nishimura N. Tsuchiya W. Moresco J. J. Hayashi Y. Satoh K. Kaiwa N.. (2018). Control of seed dormancy and germination by DOG1-AHG1 PP2C phosphatase complex *via* binding to heme. Nat. Commun. 9, 2132. doi: 10.1038/s41467-018-04437-9 29875377 PMC5989226

[B92] Nishimura N. Yoshida T. Kitahata N. Asami T. Shinozaki K. Hirayama T. (2007). ABA-hypersensitive Germination1 encodes a protein phosphatase 2C, an essential component of abscisic acid signaling in arabidopsis seed. Plant J. 50 (6), 935–949. doi: 10.1111/j.1365-313X.2007.03107.x 17461784

[B93] Oh E. Kang H. Yamaguchi S. Park J. Lee D. Kamiya Y.. (2009). Genome-wide analysis of genes targeted by PHYTOCHROME INTERACTING FACTOR 3-LIKE5 during seed germination in arabidopsis. Plant Cell 21 (2), 403–419. doi: 10.1105/tpc.108.064691 19244139 PMC2660632

[B94] Oh E. Kim J. Park E. Kim J. Kang C. Choi G. (2004). PIL5, a phytochrome-interacting basic helix-loop-helix protein, is a key negative regulator of seed germination in arabidopsis thaliana. Plant Cell 16 (11), 3045–3058. doi: 10.1105/tpc.104.025163 15486102 PMC527197

[B95] Oh E. Yamaguchi S. Hu J. Yusuke J. Jung B. Paik I.. (2007). PIL5, a phytochrome-interacting bHLH protein, regulates gibberellin responsiveness by binding directly to the GAI and RGA promoters in arabidopsis seeds. Plant Cell 19 (4), 1192–1208. doi: 10.1105/tpc.107.050153 17449805 PMC1913757

[B96] Ohtani M. Wachter A. (2019). NMD-based gene regulation - a strategy for fitness enhancement in plants? Plant Cell Physiol. 60, 1953–1960. doi: 10.1093/pcp/pcz090 31111919

[B97] Pan Q. Shai O. Lee L. J. Frey B. J. Blencowe B. J. (2008). Deep surveying of alternative splicing complexity in the human transcriptome by high-throughput sequencing. Nat. Genet. 40 (12), 1413–1415. doi: 10.1038/ng.259 18978789

[B98] Papp I. Mur L. A. Dalmadi A. Dulai S. Koncz C. (2004). A mutation in the cap-binding protein 20 gene confers drought tolerance to arabidopsis. Plant Mol. Biol. 55, 679–686. doi: 10.1007/s11103-004-1680-2 15604709

[B99] Parcy F. Valon C. Raynal M. Gaubier-Comella P. Delseny M. Giraudat J. (1994). Regulation of gene expression programs during arabidopsis seed development: roles of the ABI3 locus and of endogenous abscisic acid. Plant Cell 6, 1567–1582. doi: 10.1105/tpc.6.11.1567 7827492 PMC160544

[B100] Park J. Lee N. Kim W. Lim S. Choi G. (2011). ABI3 and PIL5 collaboratively activate the expression of somnus by directly binding to its promoter in imbibed arabidopsis seeds. Plant Cell 23 (4), 1404–1415. doi: 10.1105/tpc.110.080721 21467583 PMC3101561

[B101] Penfield S. Josse E. M. Halliday K. J. (2010). A role for an alternative splice variant of PIF6 in the control of arabidopsis primary seed dormancy. Plant Mol. Biol. 73 (1–2), 89–95. doi: 10.1007/s11103-009-9571-1 19911288

[B102] Punzo P. Ruggiero A. Possenti M. Perrella G. Nurcato R. Costa A.. (2020). DRT111/SFPS splicing factor controls abscisic acid sensitivity during seeddevelopment andgermination. Plant Physiol. 183 (2), 793–807. doi: 10.1104/pp.20.00037 32123040 PMC7271812

[B103] Qiao D. Yang C. Chen J. Guo Y. Li Y. Niu S.. (2019). Comprehensive identification of the full-length transcripts and alternative splicing related to the secondary metabolism pathways in the tea plant (Camellia sinensis). Sci. Rep. 9, 2709. doi: 10.1038/s41598-019-39286-z 30804390 PMC6389920

[B104] Raczynska K. D. Simpson C. G. Ciesiolka A. Szewc L. Lewandowska D. McNicol J.. (2010). Involvement of the nuclear cap-binding protein complex in alternative splicing in arabidopsis thaliana. Nucleic Acids Res. 38, 265–278. doi: 10.1093/nar/gkp869 19864257 PMC2800227

[B105] Reddy A. S. N. (2004). Plant serine/arginine-rich proteins and their role in pre-mRNA splicing. Trends Plant Sci. 9 (11), 541–547. doi: 10.1016/j.tplants.2004.09.007 15501179

[B106] Reddy A. S. N. (2007). Alternative splicing of pre-messenger RNAs in plants in the genomic era. Annu. Rev. Plant Biol. 58, 267–294. doi: 10.1146/annurev.arplant.58.032806.103754 17222076

[B107] Reddy A. S. N. Marquez Y. Kalyna M. Barta A. (2013). Complexity of the alternative splicing landscape in plants. Plant Cell 25, 3657–3683. doi: 10.1105/tpc.113.117523 24179125 PMC3877793

[B108] Reddy A. S. N. Rogers M. F. Richardson D. N. Hamilton M. Ben-Hur A. (2012). Deciphering the plant splicing code: Experimental and computational approaches for predicting alternative splicing and splicing regulatory elements. Front. Plant Sci. 3 (FEB). doi: 10.3389/fpls.2012.00018 PMC3355732 22645572

[B109] Reyes J. L. Chua N. H. (2007). ABA induction of miR159 controls transcript levels of two MYB factors during *Arabidopsis* seed germination. Plant J. 49, 592–606. doi: 10.1111/j.1365-313X.2006.02980.x 17217461

[B110] Rodrigues A. Adamo M. Crozet P. Margalha L. Confraria A. Martinho C.. (2013). ABI1 and PP2CA phosphatases are negative regulators of Snf1-related protein kinase1 signaling in arabidopsis. Plant Cell 25 (10), 3871–3884. doi: 10.1105/tpc.113.114066 24179127 PMC3877788

[B111] Ruan J. Guo F. Wang Y. Li X. Wan S. Shan L.. (2018). Transcriptome analysis of alternative splicing in peanut (Arachis hypogaea L.). BMC Plant Biol. 18 (1), 139. doi: 10.1186/s12870-018-1339-9 29973157 PMC6032549

[B112] Rühl C. Stauffer E. Kahles A. Wagner G. Drechsel G. Rätsch G.. (2012). Polypyrimidine tract binding protein homologs from arabidopsis are key regulators of alternative splicing with implications in fundamental developmental processesW. Plant Cell 24 (11), 4360–4375. doi: 10.1105/tpc.112.103622 23192226 PMC3531839

[B113] Saez A. Apostolova N. Gonzalez-Guzman M. Gonzalez-Garcia M. P. Nicolas C. Lorenzo O.. (2004). Gain-of-function and loss-of-function phenotypes of the protein phosphatase 2C HAB1 reveal its role as a negative regulator of abscisic acid signalling. Plant J. 37 (3), 354–369. doi: 10.1046/j.1365-313X.2003.01966.x 14731256

[B114] Sano N. Marion-Poll A. (2021). ABA metabolism and homeostasis in seed dormancy and germination. Int. J. Mol. Sci. 22, 5069. doi: 10.3390/ijms22105069 34064729 PMC8151144

[B115] Sawicka K. Bushell M. Spriggs K. Willis A. (2008). Polypyrimidine-tract-binding protein: a multifunctional RNA-binding protein. Biochem. Soc Trans. 36, 641–647. doi: 10.1042/BST0360641 18631133

[B116] Schlautmann L. P. Gehring N. H. (2020). A day in the life of the exon junction complex. Biomolecules 10, 866. doi: 10.3390/biom10060866 32517083 PMC7355637

[B117] Schwer B. Kruchten J. Shuman S. (2016). Structure-function analysis and genetic interactions of the SmG, SmE, and SmF subunits of the yeast Sm protein ring. RNA 22 (9), 1320–1328. doi: 10.1261/rna.057448.116 27417296 PMC4986888

[B118] Schwerk C. Prasad J. Degenhardt K. Erdjument-Bromage H. White E. Tempst P.. (2003). ASAP, a novel protein complex involved in RNA processing and apoptosis. Mol. Cell. Biol. 23 (8), 2981–2990. doi: 10.1128/mcb.23.8.2981-2990.2003 12665594 PMC152566

[B119] Shen H. Moon J. Huq E. (2005). PIF1 is regulated by light-mediated degradation through the ubiquitin-26S proteasome pathway to optimize photomorphogenesis of seedlings in arabidopsis. Plant J. 44 (6), 1023–1035. doi: 10.1111/j.1365-313X.2005.02606.x 16359394

[B120] Söderman E. M. Brocard I. M. Lynch T. J. Finkelstein R. R. (2000). Regulation and function of the arabidopsis ABA-INSENSITIVE4 gene in seed and abscisic acid response signaling networks. Plant Physiol. 124, 1752–1765. doi: 10.1104/pp.124.4.1752 11115891 PMC59872

[B121] Srinivasan A. Jiménez-Gómez J. M. Fornara F. Soppe W. J. J. Brambilla V. (2016). Alternative splicing enhances transcriptome complexity in desiccating seeds. J. Integr. Plant Biol. 58 (12), 947–958. doi: 10.1111/jipb.12482 27121908

[B122] Stauffer E. Westermann A. Wagner G. Wachter A. (2010). Polypyrimidine tract-binding protein homologues from arabidopsis underlie regulatory circuits based on alternative splicing and downstream control. Plant J. 64, 243–255. doi: 10.1111/j.1365-313X.2010.04321.x 20735772

[B123] Sugliani M. Brambilla V. Clerkx E. J. M. Koornneef M. Soppe W. J. J. (2010). The conserved splicing factor SUA controls alternative splicing of the developmental regulator ABI3 in arabidopsis. Plant Cell 22 (6), 1936–1946. doi: 10.1105/tpc.110.074674 20525852 PMC2910958

[B124] Suzuki M. Kao C. Y. McCartyl D. R. (1997). “The conserved B3 domain of VIVIPAROUSI has a cooperative DNA binding activity. Plant Cell 9, 799–807. doi: 10.1105/tpc.9.5.799 9165754 PMC156957

[B125] Syed N. H. Kalyna M. Marquez Y. Barta A. Brown J. W. S. (2012). Alternative splicing in plants - coming of age. Trends Plant Sci. 17, 616–623. doi: 10.1016/j.tplants.2012.06.001 22743067 PMC3466422

[B126] Tanabe N. Kimura A. Yoshimura K. Shigeoka S. (2009). Plant-specific SR-related protein atSR45a interacts with spliceosomal proteins in plant nucleus. Plant Mol. Biol. 70 (3), 241–252. doi: 10.1007/s11103-009-9469-y 19238562

[B127] Tanabe N. Yoshimura K. Kimura A. Yabuta Y. Shigeoka S. (2007). Differential expression of alternatively spliced mRNAs of arabidopsis SR protein homologs, atSR30 and atSR45a, in response to environmental stress. Plant Cell Physiol. 48 (7), 1036–1049. doi: 10.1093/pcp/pcm069 17556373

[B128] Tange T. Shibuya T. Jurica M. S. Moore M. J. (2005). Biochemical analysis of the EJC reveals two new factors and a stable tetrameric protein core. RNA 11 (12), 1869–1883. doi: 10.1261/rna.2155905 16314458 PMC1370875

[B129] Tsai A. Y. Gazzarrini S. (2012). Overlapping and distinct roles of AKIN10 and FUSCA3 in ABA and sugar signaling during seed germination. Plant Signal. Behav. 7, 10. doi: 10.4161/psb.21549 PMC3493403 22902692

[B130] Umezawa T. Sugiyama N. Mizoguchi M. Hayash S. Myouga F. Yamaguchi-Shinozaki K.. (2009). Type 2C protein phosphatases directly regulate abscisic acid-activated protein kinases in arabidopsis. Proc. Natl. Acad. Sci. U.S.A. 106, 17588–17593. doi: 10.1073/pnas.0907095106 19805022 PMC2754379

[B131] Verta J. P. Jacobs A. (2022). The role of alternative splicing in adaptation and evolution. Trends Ecol. Evol. 37 (4), 299–308. doi: 10.1016/j.tree.2021.11.010 34920907

[B132] Vlad F. Rubio S. Rodrigues A. Sirichandra C. Belin C. Robert N.. (2009). Protein phosphatases 2C regulate the activation of the Snf1-related kinase OST1 by abscisic acid in arabidopsis. Plant Cell 21 (10), 3170–3184. doi: 10.1105/tpc.109.069179 19855047 PMC2782292

[B133] Wang Z. Ji H. Yuan B. Wang S. Su C. Yao B.. (2015). ABA signalling is fine-tuned by antagonistic HAB1 variants. Nat. Commun. 6 (19), 1–12. doi: 10.1038/ncomms9138 26419884

[B134] Wang B. Tseng E. Regulski M. Clark T. A. Hon T. Jiao Y.. (2016). Unveiling the complexity of the maize transcriptome by single-molecule long-read sequencing. Nat. Commun. 7, 11708. doi: 10.1038/ncomms11708 27339440 PMC4931018

[B135] Wang Y. Wang H. Xi F. Wang H. Han X. Wei W.. (2020). Profiling of circular RNA N6-methyladenosine in moso bamboo (Phyllostachys edulis) using nanopore-based direct RNA sequencing. J. Integr. Plant Biol. 62 (12), 1823–1838. doi: 10.1111/jipb.13002 32735361

[B136] Wang X. Wu F. Xie Q. Wang H. Wang Y. Yue Y.. (2012). SKIP is a component of the spliceosome linking alternative splicing and the circadian clock in arabidopsis. Plant Cell 24 (8), 3278–3295. doi: 10.1105/tpc.112.100081 22942380 PMC3462631

[B137] Wang Y. Zhang T. Song X. Zhang J. Dang Z. Pei X.. (2018). Identification and functional analysis of two alternatively spliced transcripts of ABSCISIC ACID INSENSITIVE3 (ABI3) in linseed flax (Linum usitatissimum L.). PloS One 13, e0191910. doi: 10.1371/journal.pone.0191910 29381737 PMC5790255

[B138] Wilkinson M. Lenton J. Holdsworth M. (2005). Transcripts of vp-1 homoeologues are alternatively spliced within the triticeae tribe. Euphytica 143 (3), 243–246. doi: 10.1007/s10681-005-7856-2

[B139] Wu J. Y. Maniatis T. (1993). Specific interactions between proteins implicated in splice site selection and regulated alternative splicing. Cell 75 (6), 1061–1070. doi: 10.1016/0092-8674(93)90316-i 8261509

[B140] Xin R. Zhu L. Salomé P. A. Mancini E. Marshall C. M. Harmon F. G.. (2017). SPF45-related splicing factor for phytochrome signaling promotes photomorphogenesis by regulating pre-mRNA splicing in arabidopsis. Proc. Natl. Acad. Sci. United States America 114 (33), E7018–E7027. doi: 10.1073/pnas.1706379114 PMC5565451 28760995

[B141] Xing D. Wang Y. Hamilton M. Ben-Hur A. Reddy A. S. N. (2015). Transcriptome-wide identification of RNA targets of arabidopsis SERINE/ARGININE-RICH45 uncovers the unexpected roles of this RNA binding protein in RNA processingopen. Plant Cell 27 (12), 3294–3308. doi: 10.1105/tpc.15.00641 26603559 PMC4707455

[B142] Yang L. Liu S. Lin R. (2020). ). the role of light in regulating seed dormancy and germination. J. Integr. Plant Biol. 62, 1310–1326. doi: 10.1111/jipb.13001 32729981

[B143] Yao S. Liang F. Gill R. A. Huang J. Cheng X. Liu Y.. (2020). A global survey of the transcriptome of allopolyploid brassica napus based on single-molecule long-read isoform sequencing and illumina-based RNA sequencing data. Plant J. 103 (2), 843–857. doi: 10.1111/tpj.14754 32270540

[B144] Yoshida T. Nishimura N. Kitahata N. Kuromori T. Ito T. Asami T.. (2006). ABA-hypersensitive germination3 encodes a protein phosphatase 2C (AtPP2CA) that strongly regulates abscisic acid signaling during germination among arabidopsis protein phosphatase 2Cs. Plant Physiol. 140 (1), 115–126. doi: 10.1104/pp.105.070128 16339800 PMC1326036

[B145] Zahler A. M. Lane W. S. Stolk J. A. Roth M. B. (1992). SR proteins: A conserved family of pre-mRNA splicing factors. Genes Dev. 6 (5), 837–847. doi: 10.1101/gad.6.5.837 1577277

[B146] Zaretskaya M. V. Lebedeva O. N. Fedorenko O. M. (2022). Role of DOG1 and FT, key regulators of seed dormancy, in adaptation of arabidopsis thaliana from the northern natural populations. Russian J. Genet. 58 (7), 783–790. doi: 10.1134/S1022795422070158

[B147] Zhan X. Qian B. Cao F. Wu W. Yang L. Guan Q.. (2015). Arabidopsis PWI and RRM motif-containing protein is critical for pre-mRNA splicing and ABA responses. Nat. Commun. 6, 8139. doi: 10.1038/ncomms9139 26404089 PMC5514415

[B148] Zhang X. N. Mo C. Garrett W. M. Cooper B. (2014). Phosphothreonine 218 is required for the function of SR45.1 in regulating flower petal development in arabidopsis. Plant Signal Behav. 9, e29134. doi: 10.4161/psb.29134 25763493 PMC4203572

[B149] Zhang X. N. Mount S. M. (2009). Two alternatively spliced isoforms of the arabidopsis SR45 protein have distinct roles during normal plant development. Plant Physiol. 150 (3), 1450–1458. doi: 10.1104/pp.109.138180 19403727 PMC2705014

[B150] Zhang G. Sun M. Wang J. Lei M. Li C. Zhao D.. (2019). PacBio full-length cDNA sequencing integrated with RNA-seq reads drastically improves the discovery of splicing transcripts in rice. Plant J. 97 (2), 296–305. doi: 10.1111/tpj.14120 30288819

[B151] Zhang L. Xiang Y. Chen S. Shi M. Jiang X. He Z.. (2022a). Mechanisms of MicroRNA biogenesis and stability control in plants. Front. Plant Sci. 13. doi: 10.3389/fpls.2022.844149 PMC8957957 35350301

[B152] Zhang Q. Zhang X. Wang S. Tan C. Zhou G. Li C. (2016). Involvement of alternative splicing in barley seed germination. PloS One 11, e0152824. doi: 10.1371/journal.pone.0152824 27031341 PMC4816419

[B153] Zhang Q. Zhang W. Wei J. Gao Z. Guan J. Cui Z.. (2022b). SKIP regulates ABA signaling through alternative splicing in arabidopsis. Plant Cell Physiol. 63 (4), 494–507. doi: 10.1093/pcp/pcac014 35134199

[B154] Zhao L. Zhang H. Kohnen M. V. Prasad K. V. S. K. Gu L. Reddy A. S. N. (2019). Analysis of transcriptome and epitranscriptome in plants using pacbio iso-seq and nanopore-based direct RNA sequencing. Front. Genet. 10 (MAR). doi: 10.3389/fgene.2019.00253 PMC6438080 30949200

[B155] Zhu Z. Chen H. Xie K. Liu C. Li L. Liu L.. (2020). Characterization of drought-responsive transcriptome during seed germination in adzuki bean (Vigna angularis l.) by PacBio SMRT and illumina sequencing. Front. Genet. 11. doi: 10.3389/fgene.2020.00996 PMC7489039 33110419

[B156] Zou M. Guan Y. Ren H. Zhang F. Chen F. (2007). Characterization of alternative splicing products of bZIP transcription factors OsABI5. Biochem. Biophys. Res. Commun. 360 (2), 307–313. doi: 10.1016/j.bbrc.2007.05.226 17604002

